# Immune- and Stemness-Related Genes Revealed by Comprehensive Analysis and Validation for Cancer Immunity and Prognosis and Its Nomogram in Lung Adenocarcinoma

**DOI:** 10.3389/fimmu.2022.829057

**Published:** 2022-06-27

**Authors:** Mengqing Chen, Xue Wang, Wenjun Wang, Xuemei Gui, Zhan Li

**Affiliations:** ^1^ Department of Respiratory and Critical Care Medicine, the Affiliated Hospital of Southwest Medical University, Luzhou, China; ^2^ Department of Stem Cell and Regenerative Medicine, State Key Laboratory of Trauma, Burn and Combined Injury, Daping Hospital, Army Medical University, Chongqing, China; ^3^ Central Laboratory, State Key Laboratory of Trauma, Burn and Combined Injury, Daping Hospital, Army Medical University, Chongqing, China

**Keywords:** lung adenocarcinoma, cancer stem cell, stem cell index, immune, nomogram, muti-omics analysis, RT-PCR

## Abstract

**Objective:**

Lung adenocarcinoma (LUAD) is a familiar lung cancer with a very poor prognosis. This study investigated the immune- and stemness-related genes to develop model related with cancer immunity and prognosis in LUAD.

**Method:**

The Cancer Genome Atlas (TCGA) was utilized for obtaining original transcriptome data and clinical information. Differential expression, prognostic value, and correlation with clinic parameter of mRNA stemness index (mRNAsi) were conducted in LUAD. Significant mRNAsi-related module and hub genes were screened using weighted gene coexpression network analysis (WGCNA). Meanwhile, immune-related differential genes (IRGs) were screened in LUAD. Stem cell index and immune-related differential genes (SC-IRGs) were screened and further developed to construct prognosis-related model and nomogram. Comprehensive analysis of hub genes and subgroups, involving enrichment in the subgroup [gene set enrichment analysis (GSEA)], gene mutation, genetic correlation, gene expression, immune, tumor mutation burden (TMB), and drug sensitivity, used bioinformatics and reverse transcription polymerase chain reaction (RT-PCR) for verification.

**Results:**

Through difference analysis, mRNAsi of LUAD group was markedly higher than that of normal group. Clinical parameters (age, gender, and T staging) were ascertained to be highly relevant to mRNAsi. MEturquoise and MEblue were found to be the most significant modules (including positive and negative correlations) related to mRNAsi *via* WGCNA. The functions and pathways of the two mRNAsi-related modules were mainly enriched in tumorigenesis, development, and metastasis. Combining stem cell index–related differential genes and immune-related differential genes, 30 prognosis-related SC-IRGs were screened *via* Cox regression analysis. Then, 16 prognosis-related SC-IRGs were screened to construct a LASSO regression model at last. In addition, the model was successfully validated by using TCGA-LUAD and GSE68465, whereas c-index and the calibration curves were utilized to demonstrate the clinical value of our nomogram. Following the validation of the model, GSEA, immune cell correlation, TMB, clinical relevance, etc., have found significant difference in high- and low-risk groups, and 16-gene expression of the SC-IRG model also was tested by RT-PCR. *ADRB2*, *ANGPTL4*, *BDNF*, *CBLC*, *CX3CR1*, and *IL3RA* were found markedly different expression between the tumor and normal group.

**Conclusion:**

The SC-IRG model and the prognostic nomogram could accurately predict LUAD survival. Our study used mRNAsi combined with immunity that may lay a foundation for the future research studies in LUAD.

## Introduction

Until now, as an important branch of malignant tumors, lung cancer is still a conventional causation of tumor death (1), and about 83% of lung cancers are non–small cell lung cancer (NSCLC) (2). Lung adenocarcinoma (LUAD) is a major subtype of NSCLC, and its incidence has always been high (3).

Since targeted therapy and immunotherapy have made considerable progresses in recent years, patients with LUAD now have more chance to choose a better treatment. However, on account of lack of targeted gene mutations, low PD-L1 (CD274) expression rate, and resistance after targeted therapy, there are still a significant proportion of patients making a tumor progression and die (4). Among them, the important cause of death involves tumor growth and metastasis, and cancer stem cells (CSCs) are regarded as the key driver: CSC biology is still in its infancy, but a large amount of data shows that there was a strong correlation between the expression of stem cell–like cells and the drug resistance of lung cancer (5). This phenomenon does not only occur in patients undergoing chemotherapy, but resistance to targeted therapy may also be related to it (6–8). In addition to this, tumor cells with PD-L1 expression may occur immune escape (9). CSC can evade immune surveillance due to their immunomodulatory effects (10). CSCs also can affect the immune system, such as the immune microenvironment of tumor lymph nodes (11). However, anti-cancer therapies currently not only fail to eradicate CSC clones but also assist in the screening of resistant CSC clones from the CSC pool, leading to treatment resistance and relapse (5, 12). Moreover, with the rise of immunotherapy, opening a new era of tumor therapy may require better exploration of the interaction between the CSC and the tumor immune microenvironment (TIME) (13).

The stem cell index, also known as the stemness index, is proposed by researchers from the University of Sao Paulo to assess the degree of dedifferentiation of cancer tissues. The researchers have found that cancer stemness index have unexpected correlations with immune checkpoint expression and infiltration of immune system cells (14), and these indicators may help us identify new biomarkers. At present, many studies have used CSC index to mine new biomarkers in LUAD (15–18), but there were few research works studying the relationship between stem cell index and tumor immune infiltration and the combination of them in LUAD. Therefore, in our study, according to the definition of stem cell index, combined with the immune-related gene, using bioinformatics analysis, we screened the genes related to stem cell index and immunity, constructed the model, and verified subgroups through multi-omics aspects of bioinformatics analysis and reverse transcription polymerase chain reaction (RT-PCR), providing a new perspective for cancer immunity and prognosis of LUAD.

## Method

### Acquisition and Processing of Data

#### Getting Datasets; Survival Analysis and Clinical Correlation Analysis of Stem Cell Index

We downloaded the data from The Cancer Genome Atlas database (TCGA, https://portal.gdc.cancer.gov/), which contained transcript data of 535 tumor tissues with LUAD and 59 normal tissues (TCGA-LUAD) and clinical data of 522 patients with LUAD. We also download GSE68465 from the Gene Expression Omnibus (GEO, https://www.ncbi.nlm.nih.gov/geo/). Transcriptome profiling data of 443 tumor tissues with LUAD and 19 normal tissues in the GSE68465 dataset were used for further analysis.

Using mRNA stemness index (mRNAsi) as a variable, the R packages “survminer” was applied to analyze the correlation of mRNAsi with clinical parameters. Then, according to the median of mRNAsi, the tumor components were separate into two groups (high–mRNAsi level and low–mRNAsi level group) for survival analysis. The mRNAsi index of LUAD was acquired from the supplemental information in the study of Malta et al. (14).

#### Screening for Differential Genes and WGCNA Module Function and Pathway Enrichment Analysis

After the analysis above, we first assessed the difference between tumor and normal group according to mRNAsi, and then we used the R package “limma” (the Wilcoxon test) to screen the differential expression genes (DEGs) related to LUAD. The DEGs were next used to construct a coexpression module using a weighted gene coexpression network analysis (WGCNA). The construction process includes the following main steps: (1) give a definition for similarity matrix; (2) use the function pickSoftThreshold to select the soft threshold powerβ; (3) convert the adjacency matrix into a topological overlap matrix (TOM); (4) execute hierarchical aggregation of dissTOM derived from TOM; (5) from the hierarchical clustering tree, use the dynamic tree cutting method to distinguish modules with identical expression profiles; (6) quantify the coexpression similarity of the entire modules and compute their characteristic genes, etc. (19). At last, we selected two modules with the highest absolute value associated with mRNAsi (including positive and negative correlations) for the following analysis.

For better understanding the functions and pathways of the two mRNAsi-related modules above in LUAD, each of them was analyzed for Gene Ontology (GO) and Kyoto Encyclopedia of Genes and Genomes (KEG)G enrichment, respectively. R package “colorspace”, “stringi”, and “ggplot2” were used. The GO enrichment analysis included three components: molecular function (MF), cellular component (CC), and biological process (BP). Choose the threshold as p-value <0.05 and q-value <0.05.

#### Intersection of Stem Cell Index–Related Differential Genes and Immune-Related Differential Genes and Univariate Cox Regression Analysis and Construct LASSO Regression Model

To discover the immune-related genes (IRGs) in LUAD, we first downloaded the IRG data from the immunology database and analysis website (ImmPort, https://www.immport.org/). By taking the intersection with the DEGs that we screened before, we extracted the LUAD immune-related DEGs for the next step. We further analyzed the intersection of mRNAsi-related DEGs and immune-related DEGs *via* Venn diagram.

Using the R package “survival”, we further screened for prognosis-related hub genes by univariate Cox regression analysis. We selected the genes with P < 0.05 and HR ≠ 1 from the univariate Cox analysis. The Least Absolute Shrinkage and Selection Operator (LASSO) regression analysis is a popular algorithm, which was extensively utilized in medical studies (20, 21). Using the R package “glmnet” and “survival”, the optimal model based on prognosis-related stem cell index–related differential genes and immune-related differential genes (SCIRGs) was subsequently identified utilizing LASSO regression analysis (22). The model formula is


$$Riskscore=\sum_{\text{i}=1}^{\text{n}}\text{(Coefi}\times \text{Ni)}$$

where Coef refers to the regression coefficient of SCIRGs in LASSO Cox regression analysis, “Ni” is the expression value of the gene, and “n” is the number of SCIRGs.

#### Verify the Risk Score Model Based on SCIRGs and Construct a Prognostic Nomogram

To verify the predictive ability of the model, we assessed the model through the training set (TCGA-LUAD) and the validation set (GSE68465), respectively. Using R package “survival” and “survminer” for survival analysis, we drew a Kaplan–Meier curve in TCGA and GEO datasets, separately. To explore high- and low-risk hub genes in the model and the risk score distribution in LUAD, we used the “pheatmap” package to depict risk curves, survival status maps, and risk heat maps. In addition, using R package “survival” for an independent prognostic analysis of the training and validation set, these helped us to understand whether the risk score can be used as a prognostic factor independent of clinical parameters. We used R package “survivalROC” to draw a multi-index ROC curve to assess prediction accuracy of the model. Last, we further take risk score with clinical parameters to draw a nomogram. The clinical parameters included age, gender, TNM (TNM Classification of Malignant Tumors, UICC 8th edition), and stage. The nomogram was used to evaluate the 1-, 2-, and 3-year survival rates of patients. The predictive capability of the model was assessed by calculating the C-index and plotting the calibration curves.

#### Comprehensive Analysis of Molecular and Subgroups Characteristics in the Model

Gene set enrichment analysis (GSEA) and the frequency of gene mutations were analyzed in high- and low-risk groups by utilizing the Maftools package of R. Furthermore, the association of high- and low-risk groups with TIME was also validated. CIBERSORT (https://cibersort.stanford.edu/) was used to input the data and perform 1,000 iterations to explore the 22 immune cells’ proportions. In addition to this, we compared the difference of 22 immune cells’ related function between the two subgroups. The correlation between risk score and common oncogene (*EGFR*, *ALK*, *ROS1*, *KRAS*, and *TP53*), *CD274*, and stem cell index (DNAss and RNAss) were also explored. Then, correlation analysis was performed between tumor mutation burden (TMB) and risk score, and the difference analysis between TMB and the subgroups was also explored. We also explored the distribution of every samples classified by clinical parameters between the subgroups, and the difference of stage and immunophenotyping was further demonstrated. Finally, the drug sensitivity analysis of every hub gene was demonstrated using CellMiner.

To gain a deep understanding of the key genes in the model, we use Oncomine (https://www.oncomine.org), UALCAN (http://ualcan.path.uab.edu), Kaplan–Meier (http://kmplot.com), TIMER (https://cistrome.shinyapps.io/timer/), GEPIA (https://gepia.cancer-pku.cn), and other web-based bioinformation tools to perform differential analysis, survival analysis, immune infiltration analysis, and correlation analysis in LUAD. In the correlation analysis, so as to represent the strength of the interrelationship between gene expression and tumor immune infiltration in TIMER, we categorized it as follows: 0.00–0.19, “very weak”; 0.20–0.39, “weak”; 0.40–0.59, “moderate”; 0.60–0.79, “strong”; and 0.80–1.0, “very strong”.

### Cell and Stem Cell Culture

We purchased human bronchial epithelial cells (Beas-2B) and human LUAD cell lines (A549 and HCC827) from American Type Culture Collection (USA). Beas-2B was cultured with Dulbecco’s modified Eagle medium (DMEM) containing 10% fetal bovine serum (Gibco; Thermo Fisher Scientific, USA). We utilized RPMI-1640 medium (Biological Industries, Israel) with 10% fetal bovine serum to sustain A549 and HCC827 cell lines. Further, we cultured cells at 37°C with an atmosphere of 5% CO2. Then, the pretreated cells (A549 and HCC827) were suspended in DMEM/F12 medium and added with 20 ng/ml EGF (Sigma), 20 ng/ml bFGF (BD Biosciences), and 2% B27 (Gibco; Thermo Fisher Scientific, USA) to further study stem cells. The mRNA expression levels of SCIRGs in the model were detected by RT-PCR.

### Quantitative Real-Time Polymerase Chain Reaction

We take the cell line with a good growth status, using TRIzol reagent for total RNA extraction, and further transcribed into cDNA by reverse transcription. RT-PCR was performed using the SYBR qPCR mix (Takara Bio Inc) in the 7500 real-time PCR system (Thermo Fisher Scientific). Glyceraldehyde-3-phosphate dehydrogenase (GADPH) was selected as the standardized endogenous reference. See **Supplementary Table 1** for the primer sequences of GAPDH and SCIRGs in the model.

### Statistical Analysis

All analyses were performed using R version 4.1.1. The purpose of every statistical analysis was described in the specific section in Method. Experiments in this study were performed in triplicate with the statistical results presented as means ± standard deviation (SD) using GraphPad Prism Software (version 9.3, CA, USA). Student t-test was applied to compare the differences between the two groups. Differences were considered statistically significant if the p-value was < 0.05.

## Results

### Routinely Analyze the Characteristics of mRNAsi in LUAD

**Figure 1:**
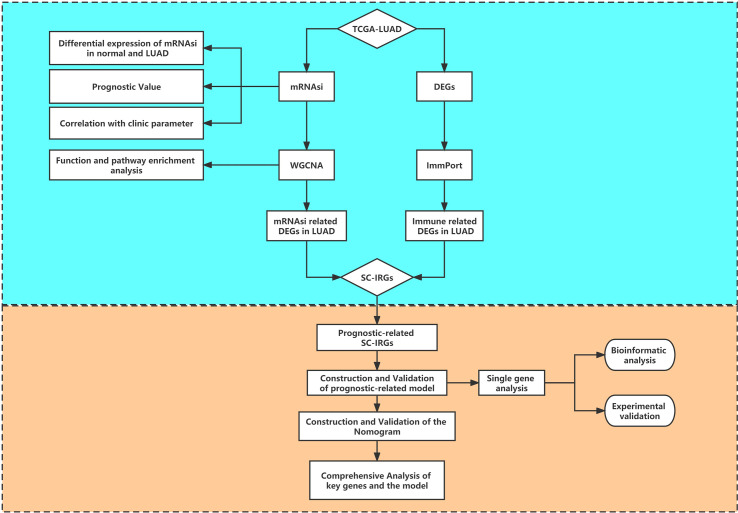
Flow diagram of the study.

**Figure 1** provides a flow blueprint of the analysis process. The overall process is mainly divided into method development, SC-IRGs screening, model validation, and key gene identification.

**Figure 2:**
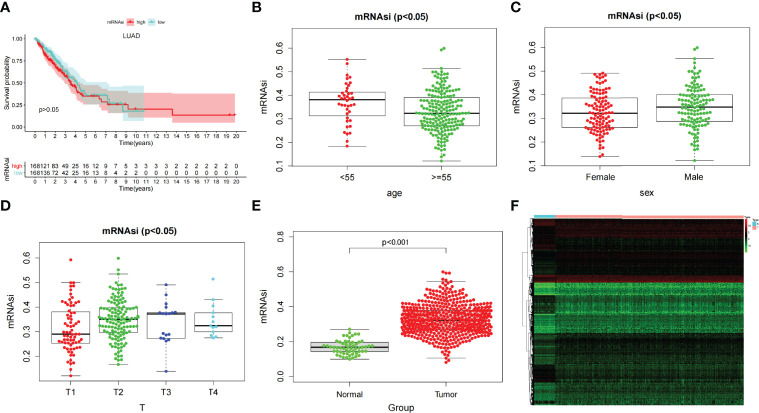
**(A)** Kaplan–Meier displays no significant difference between the high- and low-mRNAsi groups. **(B–D)** The correlation of global mRNAsi profiles with LUAD clinical subtypes: **(B)** age, **(C)** sex, and **(D)** T staging. **(E)** Different analysis of the mRNAsi level between normal and LUAD tissues. **(F)** The heat map of DEGs in LUAD.

After dividing tumor group into two subgroups according to the median (high–mRNAsi level and low–mRNAsi level groups), survival analysis did not show any considerable difference between them (p > 0.05) (**Figure 2A**). This suggested that we needed to explore the significance of mRNAsi in LUAD from other perspectives. We then mined the correlation of mRNAsi with clinical parameters (age, sex, and TNM). The results exhibited that the mRNAsi level of the group that was younger than 55 years old was higher than that of the group which was greater than 55 years old (p < 0.05) (**Figure 2B**); the mRNAsi level of the male was higher than that of the female (p < 0.05) (**Figure 2C**). Moreover, in terms of tumor stages, the mRNAsi level was markedly different in T stages (p < 0.05), and it showed a gradually increasing trend (**Figure 2D**). The relationship between mRNAsi and clinical factors laid the foundation for us to further screen for genes related with mRNAsi.

### Most Significant Modules of mRNAsi *via* WGCNA and Module Function and Pathway Enrichment Analysis

**Figure 3:**
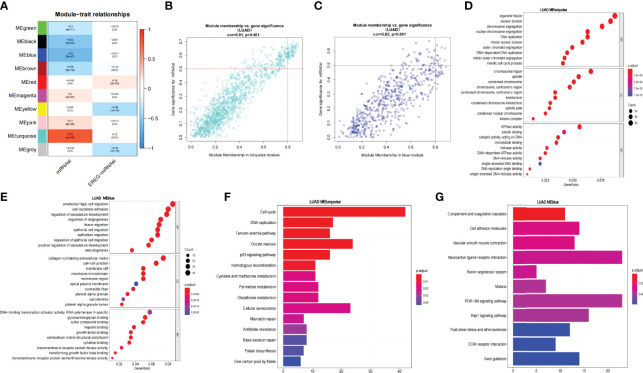
WGCNA of LUAD and enrichment analysis of the significant modules. **(A)** Correlation of the gene module with mRNAsi and EREG-mRNAsi. **(B, C)** Scatter graph of the blue module (module membership vs. gene significance). Scatter graph of the turquoise module (module membership vs. gene significance). **(D, E)**. GO enrichment analysis of the blue and turquoise modules. **(F, G)** KEGG pathway enrichment analysis of the blue and turquoise modules.

Through difference analysis, we found that the mRNAsi level in the tumor group was markedly higher than in normal group (p < 0.001), and then the DEGs were screened out for the following analysis (**Figures 2E, F**). WGCNA was further executed on DEG to sort out gene coexpression modules. The power β = 3 was used to determine a scalefree topology index (R^2^) of 0.97, and dynamic hierarchical tree cutting algorithm was adopted to detect coexpression module (**Supplementary Figures 1A–C**). Ten modules were obtained in mRNAsi (**Figure 3A**). MEturquoise (R = 0.78, p < 0.001) and MEblue(R = −0.6, p < 0.001) had the most significant correlations with mRNAsi, and we finally selected genes whose module membership was greater than 0.8 and gene significance for mRNAsi was greater than 0.5 in the two modules for further analysis (**Figures 3B, C**).

Then, we analyzed the function and pathway enrichment of two modules, respectively. In GO enrichment analysis, it was found that the MEturquoise module more participated in tumor growth and reproduction than MEblue module. For example, in BP, the MEturquoise module was enriched in chromosome segregation, nuclear division, nuclear chromosome segregation, DNA replication, etc. In CC, MEturquoise module was enriched in chromosome region, spindle, condensed chromosome, etc. In MF, MEturquoise module was enriched in ATPase activity, tubulin binding, microtubule binding, DNA replication origin binding, etc. Whereas MEblue module was mainly enriched in tumor microenvironment such as vasculogenesis and may have some relationship in tumor metastasis (**Figures 3D, E**). Similarly, KEGG pathway enrichment analysis exhibited that in the MEturquoise module, genes were related to cell cycle, DNA Replication, p53 signaling pathway, cell senescence, mismatch repair, and base excision repair; whereas in the MEblue module, genes were enriched in cell adhesion molecules and vascular smooth muscle contraction, which may play an important role in tumor metastasis (**Figures 3F, G**).

### Screening for Prognosis-Related SCIRGs and Univariate COX Regression Analysis and Construct Model

**Figure 4:**
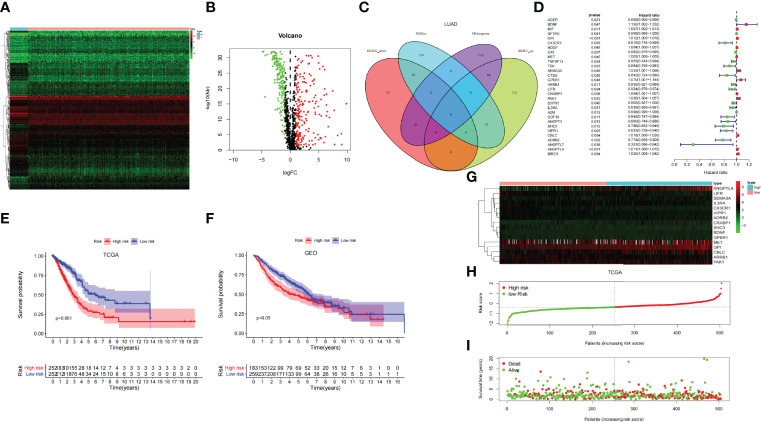
**(A)** The heat map of immune-related DEGs in LUAD. **(B)** Volcano map of immune-related DEGs in LUAD. Green, downregulated genes; red, upregulated genes. **(C)** Venn diagram of the intersection genes related to both mRNAsi and immunity. **(D)** Univariate COX regression analysis of prognosis-related stem cell and immune-related differential genes (SCIRGs) in LUAD. **(E, F)** Kaplan–Meier curves show a considerable difference between the high- and the low-risk groups. **(G)** Heat maps of the hub genes’ expression pattern, where the red to green means changes from high to low expression in TCGA. **(H)** Distribution of multi-genes signature risk score in TCGA datasets. **(I)** The survival status and interval of TCGA-LUAD patients.

Through the heat map and volcano map (**Figures 4A, B**), we found that in LUAD, there were 359 immune-related DEGs, including 168 downregulated genes and 191 upregulated genes. Then, we intersected these upregulated and downregulated immune-related DEGs with MEturquoise and MEblue modules, respectively. The intersection genes related to both mRNAsi and immunity were obtained (**Figure 4C**). Among them, the intersection of MEturquoise module and immune downregulated genes (IRDEG_down) contained 26 genes, whereas the intersection of MEturquoise module and immune upregulated genes (IRDEG_up) got 48 genes. The intersection of MEblue module and immune downregulated genes (IRDEG_down) contained 69 genes, whereas the intersection of MEblue module and immune upregulated genes (IRDEG_up) got 11 genes. The 154 genes were used for sorting out prognosis-related SCIRGs further.

Through univariate COX regression analysis, we sorted out the prognosis-related SCIRGs among the intersection genes. The HR of *ANGPTL7*, *ADRB2*, *SHC3*, *CX3CR1*, *VIPR1*, *CTSG*, *GDF10*, *ANGPT1*, *TEK*, *LIFR*, *IL3RA*, *TNFSF13*, *ARRB1*, *S1PR1*, *CAT*, *AGER*, *A2M*, and *SFTPD* were <1, which indicated that those genes were low-risk genes; whereas for the HR of *MET*, *HDGF*, *CRABP1*, *MIF*, *ANGPTL4*, *GPI*, *CBLC*, *BIRC5*, *PAK1*, *SEMA3A*, *GPER1*, and *BDNF*>1, it indicated that those genes were high-risk genes (**Figure 4D**). Furthermore, LASSO regression was executed to select the optimal predictive factors (genes), preventing overfitting, and then to build a LASSO Cox regression model.

We finally got 16 genes to construct LASSO Cox regression model (Supplementary **Figures 1D, E**). The formula for the model is as follows: risk score = 0.02733 * *BDNF* + 0.004734 * *GPI* + (−0.05939) * *CX3CR1* + 0.00120 * *MET* + 0.00960 * *SEMA3A* + 0.00503 * *GPER1* + (−0.00995) * *ARRB1* + (−0.02840) * *LIFR* + 0.00206 * *CRABP1* + 0.00804 * *PAK1* + (−0.02285) * *IL3RA* + (−0.05521) * *SHC3* + (−0.00924) * *VIPR1* + 0.00051 * *CBLC* + (−0.02154) * *ADRB2* + 0.00719 * *ANGPTL4 (* 23).

### Validation of the Model and Construction and Validation of the Nomogram

To demonstrate whether the final model was robust in different populations, we singled out a cutoff value in the internal training set (TCGA-LUAD) and performed an identical formula in external validation set (GSE68465). According to the median risk value in the TCGA dataset, patients were separated into high-risk groups and low-risk groups. Comparing with the low-risk group, the high-risk group showed a better prognosis both in the TCGA and GEO datasets (**Figures 4E, F**).

Heat map shows that the expressions of *ANGPTL4*, *GPI*, *CBLC*, and *PAK1* are higher in the high-risk group than in the low-risk group, regardless of the training set or the validation set, which pointed out that they may be carcinogenesis. On the contrary, in the low-risk group, the expressions of *IL3RA*, *CX3CR1*, *ARRB1*, *LIFR*, and *VIPR1* were higher than those in the high-risk group, which signified that they have a tumor suppressor effect. Risk curves and survival status maps showed same trends in TCGA and GEO, patients with higher scores were more likely to have a poorer prognosis (**Figures 4G-I**; **Supplementary Figure 2**).

**Figure 5:**
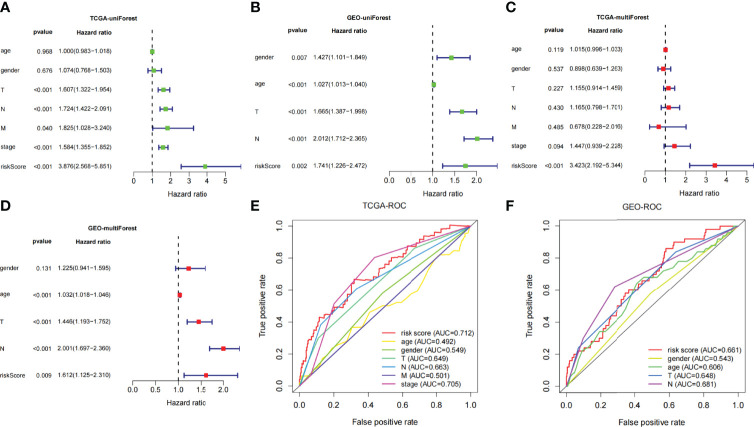
**(A, B)** Univariate Cox regression analyses of overall survival in TCGA and GEO dataset. **(C, D)** Multivariate Cox regression analyses of overall survival in TCGA and GEO dataset. **(E, F)** Comparing the AUCs of the risk scores with other clinical parameters in TCGA and GEO dataset.

Univariate- and multivariate-independent prognostic analyses were carried out to explore the correlation between prognosis and clinical parameters and risk score and verified in the TCGA and GEO dataset, respectively. Through univariate-independent prognostic analysis, in the TCGA dataset, the clinical parameters T, N, and M staging, stage, and risk score were associated with prognosis; whereas in GEO dataset, gender, age, T and N staging, and risk score were related to prognosis (**Figures 5A, B**). Through multivariate-independent prognostic analysis, it revealed that in the TCGA dataset, risk score was related to prognosis; whereas in the GEO validation set, T and N staging and risk score were associated with prognosis (**Figures 5C, D**). These indicated that risk score was independent of clinical parameters to be a prognostic parameter. To go step further, we assessed the prediction accuracy of the model through ROC curve. The areas under curves (AUCs) of the risk score were 0.712 in TCGA and 0.661 in GEO dataset, respectively. Comparing with other clinical parameters, the model had the largest value of AUC in TCGA dataset; whereas in GEO dataset, it also had the second largest value of AUC except for the N Staging (**Figures 5E, F**). This indicated that the risk score may be a better parameter with better sensitivity and specificity for predicting prognosis.

**Figure 6:**
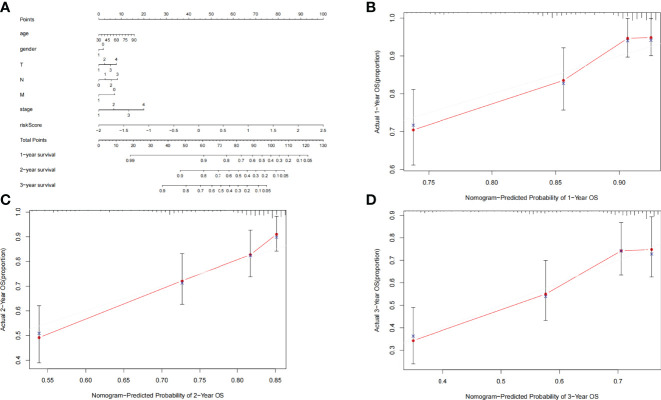
**(A)** Nomogram was assembled by clinical parameters and risk signature for predicting survival of patients with LUAD. **(B)** One-year nomogram calibration curves. **(C)** Two-year nomogram calibration curves. **(D)** Three-year nomogram calibration curves.

For the convenience of application, we have constructed a nomogram. Age, gender, TNM staging, stage, and risk score were utilized as predictive parameters to construct the nomogram (24), and we calculated the total points to obtain the 1-, 2-, and 3-year overall survival in LUAD (**Figure 6A**). Furthermore, for the accuracy of the model, we used the consistency index (C-index) and calibration curve to estimate. The C-index was 0.699 (0.649–0.749). The horizontal and vertical coordinates of every calibration curves represented the predicted probability and actual probability of every year overall survival (**Figures 6B–D**). The results of the calibration graph exhibited that the nomogram has a good capability to foresee the overall survival rate of patients with LUAD.

### Comprehensive Analysis of Gene and Immune Characteristics in the Model

#### Immune Characteristics of the Key Genes and the Model

**Figure 7:**
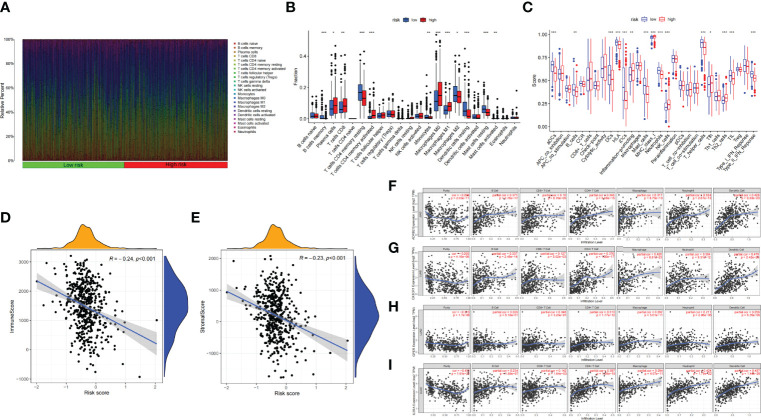
**(A)** Bar plot presents the distribution of 22 kinds of TICs in LUAD tumor samples. Column names represent sample ID. **(B)** Bar plot presents the difference of TICs between the high- and low-risk groups. **(C)** Bar plot presents the difference of immune-related function between the high- and low-risk groups. **(D, E)** Association between tumor immune microenvironment (TIME) and risk score. **(D)** ImmuneScore; **(E)** StromalScore. **(F, I)** TIMER: Immune correlation analysis of SCIRGs in the model based on immune infiltration, **(F)** *ADRB2*, **(G)** *CX3CR1*, **(H)** *GPER* (*GPER1*), and **(I)** *IL3RA.* *p < 0.05, **p < 0.01, ***p < 0.001.

The infiltration proportion of every immune cell in the two risk groups is shown, respectively (**Figure 7A**). Plasma cells, CD8 T cells, activated memory CD4 T cells, M0 and M1 macrophages, and activated mast cells were more abundant in the high-risk subgroup; correspondingly, immune-related function like inflammation-promoting, MHC class I, NK cells, and Tfh were more frequency in the high-risk subgroup. Whereas memory B cells, memory resting CD4 T cells, monocytes, M2 macrophages cell, resting dendritic cells, resting mast cells, etc., were more abundant in the low-risk subgroup; correspondingly aDCs, B cells, DCs, HLA, mast cells, etc., were more common in the low-risk group (**Figures 7B, C**). The association of risk score with TIME shows that both immune score and stromal score were negatively relevant to risk score (**Figures 7D, E**).

Furthermore, to prove the relevance of these genes to immunity, we compared the correlation between hub genes and immune cells through TIMER. *ADRB2* has moderate correlation with dendritic cell; *CX3CR1* has moderate correlation with macrophage and neutrophil; IL3RA also has moderate correlation with neutrophil and dendritic cell. Apart from these genes, other genes also have weak correlation with immune cell (**Figures 7F–I**; **Supplementary Figure 3**).

#### Clinical Characteristics of the Key Genes and the Model

**Figure 8:**
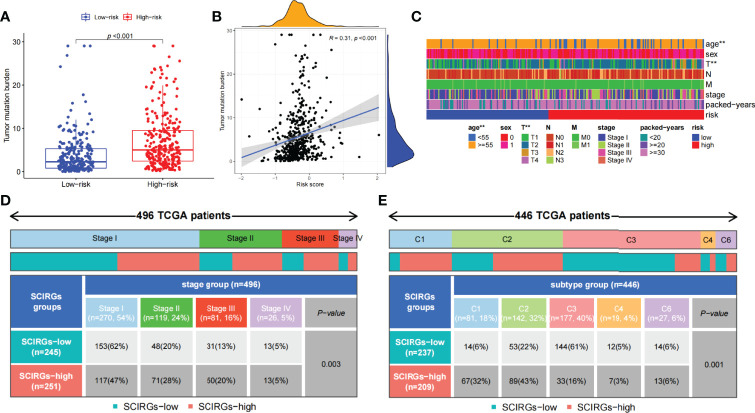
**(A)** The difference of TMB between the high- and low-risk groups. **(B)** Association between TMB and risk score. **(C)** The proportion of clinical characteristics of every sample in relative risk group was presented in heat map. **(D)** Proportion of patients in different stages of high- and low-risk groups. **(E)** Proportion of patients in different immune sub-typing of high- and low-risk groups. *p < 0.05, **p < 0.01, ***p < 0.001.

The relationship between risk score and TMB was further probed. The results exhibited that TMB was markedly higher in the high-risk group than in the low-risk group, and the higher the risk score, the larger the TMB (R = 0.31, p = 4e−12; **Figures 8A, B**). To explore the difference of every samples classified by clinical parameters between those two groups, clinical relevance heat map was used. In **Figure 8C**, age and T staging have markedly difference between the two groups. We also found that the proportion of stage IV samples has almost equal distributions between the two groups, and there were more samples in the high-risk subgroup and fewer samples in the low-risk subgroup in stages II–III, but there was an opposite result in stage I (p = 0.003, chi-square test) (**Figure 8D**). Then, 446 TCGA samples were further classified according to immune subtype. As shown in **Figure 8E**, there were more C3 subtypes in the SCIRG-low subgroup, whereas more C1 and C2 subtypes in SCIRG-high subgroup (p = 0.001, chi-square test).

Finally, the SCIRG gene was analyzed in combination with drug sensitivity, and the first 16 drugs with statistically significant differences were selected. The results uncovered that the expression level of *CX3CR1* was positively relevant to the sensitivity of Alectinib, LDK-378, Denileukin Diftitox Ontak, Estramustine, Nelfinavir, PF-06463922, and Carmustine. This indicated that the higher the expression of *CX3CR1*, the stronger the sensitivity to the abovementioned drugs. We also ascertained that the *CX3CR1* expression level was negatively relevant to the sensitivity of Irofulven. In addition, *CRABP1* expression level was positively relevant to the sensitivity of Bendamustine and Dexrazoxane. The expression of *MET* was negatively relevant to the sensitivity of Bendamustine and Dexrazoxane. The expression of *LIFR* and *BDNF* was negatively relevant to the sensitivity of Tamoxifen, whereas the expression of *GPER1* was positively relevant to Procarbazine (**Supplementary Figure 4**).

#### General Characteristics of the Key Genes and the Model

We then implement GSEA analysis to find out in which function the two subgroups of genes were up- or downregulated. For example, the genes of the high-risk group were upregulated in chromosome segregation, cornification, DNA dependent, DNA replication, and epidermal cell differentiation, whereas they also upregulated in cell cycle, DNA replication, proteasome, pyrimidine metabolism, and spliceosome in KEGG. This tells us that the high-risk group was mainly correlated with proliferation in LUAD. On the other hand, the genes of the low-risk group were downregulated in cilium movement, rDNA heterochromatin assembly, ciliary plasma, cilium, and DNA packaging complex in GO, whereas they also have the same performance in asthma, Fc epsilon RI signaling pathway, long-term depression, systemic lupus erythematosus, and vascular smooth muscle contraction in KEGG (**Supplementary Figures 5A–D**, p < 0.05). Next, we analyzed gene mutations to gain further insight in the charicteristics of the subgroups. We found 96.03% samples were altered in high-risk groups, whereas 80.58% samples were altered in low-risk groups. Missense variations were the most common mutation type (**Supplementary Figures 5E, F**). In addition to this, the risk score was also markedly relevant to common oncogenes expression, such as *ALK*, *ROS1*, *KRAS*, and *TP53*, but no markedly relevant to CD274 (**Supplementary Figure 6**).

Here, to find out potential biomarkers in LUAD, we explored the 16 genes in the model through bioinformatics. First, Oncomine was used to explore the overall difference of the above genes in lung cancer, and UALCAN was utilized to seek every gene differential expression between LUAD and normal. As shown in **Supplementary Figures 7**, **8**, whether in Oncomine or in UALCAN, *ADRB2*, *ARRB1*, *BDNF*, etc., had a lower expression in tumor group than normal tissues, whereas *CBLC*, *GPI*, and *PAK1* had a higher expression in tumor group than normal tissues. There were no studies of *ANGPTL4*, *CRABP1*, *MET*, and *SEMA3A* in Oncomine, but in UALCAN, they were frequently expressed in tumor group than normal tissues. Second, the survival analysis of key genes in LUAD was analyzed by the Kaplan–Meier method. The results exhibited that the high expression groups of *ANGPTL4*, *CBLC*, *CRABP1*, etc., have a poorer prognosis in LUAD than the low expression group, whereas the survival analysis of *ADRB2*, *CX3CR1*, *GPI*, etc., in LUAD exhibited that the prognosis was better in the high expression group (**Supplementary Figure 9**). To further understand the correlation between these key genes and common oncogenes such as *TP53*, *EGFR*, and *CD274*, we explored the correlation through GEPIA (spearman, P value < 0.05 and R > ± 0.1). Genes related to TP53 include *ANGPTL4*, *ARRB1*, *CBLC*, etc.; genes related to *EGFR* include *ADRB2*, *ANGPTL4*, *ARRB1*, etc; genes related to *CD274* include *ADRB2*, *BDNF*, *CBLC*, etc. (**Supplementary Figures 10**–**12**).

#### Experimental Verification of the Key Genes

**Figure 9:**
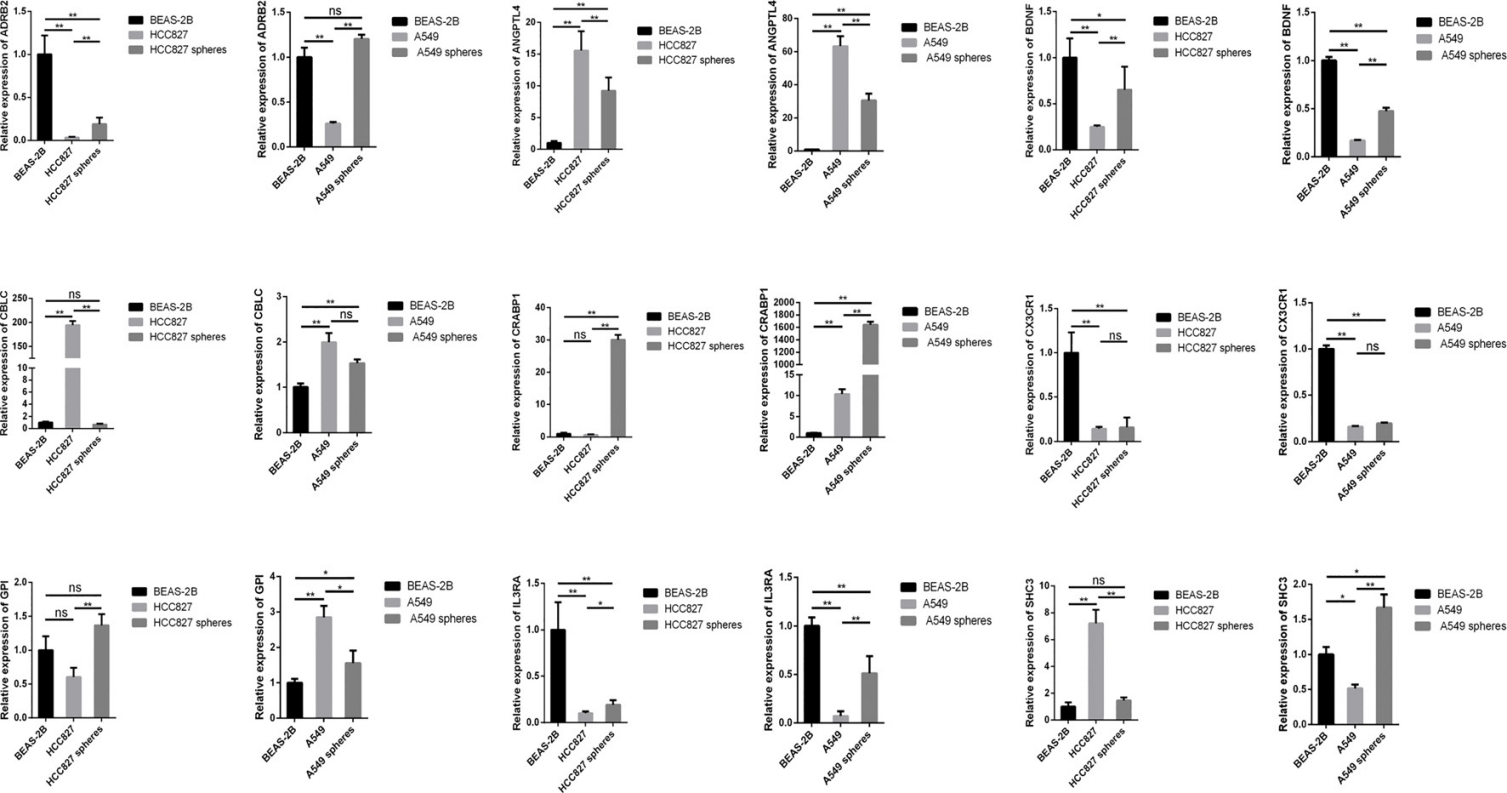
The expression levels of SCIRGs in the model between Beas-2B, HCC827 cell lines, HCC827 cancer stem cell, and results of the RT-PCR to determine gene expression. *p < 0.05, **p < 0.01, ***p < 0.001; ns, no significance.

We respectively compared the expression of the SCIRGs in normal lung epithelial cells, lung cancer cells, and lung CSCs and repeatedly compared them in different cell lines (A549 and HCC827) (**Figure 9**; **Supplementary Figure 13**). The results showed that in the A549 cell line, the expression results of nine genes in the 16 genes were consistent with those in UALCAN (**Figure 9**; **Supplementary Figures 8**, **13**): *ADRB2*, *ANGPTL4*, *BDNF*, *CBLC*, *CRABP1*, *CX3CR1*, *GPI*, *IL3RA*, and *SCH3*; in the HCC827 cell line, the expression results of nine genes among the 16 genes were consistent with those in UALCAN: *ADRB2*, *ANGPTL4*, *BDNF*, *CBLC*, *CX3CR1*, *IL3RA*, *LIFR*, and *MET*. Therefore, half or more of the genes in our model were consistent with the gene expression results of external data.

## Discussion

Since De Maria et al. have found that the *CD133* undifferentiated cells in LUAD can produce tumor xenografts that have the same phenotype with the primary LUAD in mmunodeficient mice, more and more studies began to identify lung CSC-related biomarkers and explore the characteristics of stem cells in growth, reproduction, metastasis, drug resistance of lung caner, etc. (5, 25). In addition to the discovery that *CD133* and *ALDH1* can be as biomarkers of lung CSCs, there were many explorations on the self-renewal, metabolism, drug resistance of LUAD stem cells, and even gene expression profile analysis (26–29). The ability to produce differentiated cells and to self-renew was the characteristic of stem cells, and stemness was defined as the potential for self-renew and differentiation from the cell of origin (30). To define signatures to quantify stemness and to estimate the degree of carcinogenic dedifferentiation, previous studies utilized a set of logistic regression machine learning algorithms (OCLR) to generate a stemness index (14). In recent years, its significance had been confirmed by the bioinformatics analysis in various tumors (31, 32), which also included the stemness indices of LUAD (15–18). However, few studies have combined stemness indices and immunity to construct models and explore stem cell index and immune-related differential genes in LUAD. In recent years, tumor microenvironment infiltration and tumor immunotherapy have played an important role in LUAD (33, 34). Therefore, we combined stemness indices and immune-related differential genes to construct model and explore the significance of these genes in LUAD.

Throughout the current research on mRNAsi, many studies have found the difference of mRNAsi between tumor and normal group in NSCLC. The difference analysis, survival analysis, and clinical correlation analysis of mRNAsi also have certified that mRNAsi was indeed markedly higher in tumor group than in the normal group, and it has a certain correlation with various clinical parameters in LUAD. The module that contained the highest correlation with mRNAsi was found through WGCNA and finally found and verified hub genes of LUAD. Some studies further combined the key genes with clinical parameters to construct models to help predict prognosis (15–18). Previous studies have found that the stemness was a crucial part in anti-cancer immunity (35), but the abovementioned studies did not combined mRNAsi with immunity nor did it explore the high- and low-risk groups in the model. In addition, although some of the studies selected clinical samples for verified the model, they neither explain the subgroups characteristic in the model nor construct a nomogram. As the correlation between tumor prognosis, treatment and immunity have been demonstrated by current tumor immunotherapy, there was still a need for research to explore the relationship and mechanism between immunity and tumors. Therefore, in view of previous studies of mRNAsi in LUAD, our study combined mRNAsi with IRGs from the current immune database ImmPort, intersected the mRNAsi-related modules obtained by WGCNA with immune-related differential genes, finally obtained the SCIRGs. In the subsequent construction and verification of model, the previous studies did not carry out internal and external verification of the model. In contrast, our study used the TCGA and GEO datasets for internal and external verification, and the consistency of internal verification and external verification provided a reliable basis for the application of our model. We found risk score was regarded as a risk factor both in the risk curve of **Figure 6** and independent prognostic analysis of **Figure 7**, but when using Kaplan–Meier to verify the survival analysis of the model, we found that the prognosis of the high-risk group was better than the low-risk group no matter in the internal or external datasets. This suggested that it may have other factors that affect the prognosis of patients with risk score. The potential mechanism is worthwhile for further discussion in the future. In addition, we further explored the differences in function and enrichment, gene mutation frequency, immune cell type, immune-related function, and TMB and explored the differences of clinical features in the high- and low-risk groups. As a result, more immune-related functions were higher in the low-risk group than in the high-risk group, the risk score was negatively relevant to tumor immunity and positively relevant to tumor mutation burden. The relevant mechanisms of this phenomenon can be further explored in the future. As there were few similar studies at present, our study was enriched for the research on stem cell index combined with immunity in LUAD and confirmed the conclusions of previous studies. Incidentally, mining the correlation between classic oncogenes and immune genes and risk scores exploring the situation of each key gene in the model were also the difference between our study and current study.

For the key genes in the model, *c-Met* that is a part of *RTKs* family is a known CSC marker in previous study (36). *Met* and its ligand, *HGF*, were core roles in signaling pathways of the oncogenic process, which was included the regulation of angiogenesis, cell proliferation, invasion, and CSC regulation (37). In addition, in the previous study of NSCLC, *MET* amplification was particularly related to the inflammatory microenvironment, indicating that MET-amplified tumor might respond to ICIs (38). Over the years, previous studies have found and well replicated the roles of neurotrophins in tumor development. In particular, it was reported that nerve growth factor (*NGF*) and brain-derived neurotrophic factor (*BDNF*) could stimulate tumor cell proliferation, survival, migration, and/or invasion and was beneficial to tumor angiogenesis (39). Adrenergic receptors (*ARs*), especially *β-ARs*, are expressed in most mammalian cells and relevant to kinds of malignancies including lung cancer (40). *ADRB2* encodes β-2-adrenergic receptor. Previous study has found that Beta2-AR was highly expressed in both LUAD and LUSC but clearly highly expressed in LUAD when compared with LUSC and with their matched surrounding non-tumor tissue (41). In addition, the cross-talk between macrophages and cancer cells through *CX3CR1* and *CCR2* is the basic mechanism resulting to lung cancer (42). The knockdown of *PAK1* hinders the proliferation and invasion of NSCLC (43). *ANGPTL4* was relevant to NSCLC progression and regulated epithelial-mesenchymal transition *via* ERK pathway, indicating that *ANGPTL4* is vital for the proliferation and metastasis of lung cancer, and may regard as a brand-new target for the treatment of lung cancer (44). There are many studies showing the significance of key genes in our model in LUAD or CSC. Our RT-qPCR results found that even for the key genes in the model, there were significant differences of many gene expressions between CSCs and cancer cells. We speculate that the difference was related to the underlying mechanisms of CSCs. Moreover, we ascertained that the different expression of *ADRB2*, *ANGPTL4*, *BDNF*, *CBLC*, *CX3CR1*, and *IL3RA* in tumor and normal group was consistent both in PCR and UALCAN. Combined with the previous analysis, it is indispensable to further analyze the underlying system of CSCs and the above genes in lung cancer in future research.

## Conclusions

Our research explored genes to construct the current model from the perspective of combining stem cell index and immunity and analyzed and verified the model *via* multi-omics analysis. At the same time, it verified the characteristics of genes in the model through bioinformatics analysis and experiments. However, our study neither analyzes the mechanism of CSC through laboratory methods nor explores the mechanism of genes in the model in lung cancer through experimental methods. In addition, the robust of the prognostic model required more clinical samples and experiments for demonstration. In the future, more research studies are needed to explore from the above directions.

## Data Availability Statement

The datasets analyzed during the current study are available in the TCGA, GEO under the accession number GSE68465, Oncomine, TIMER, UALCAN, Kaplan–Meier Plotter, and GEPIA.

## Author Contributions

Conception and design of the work: MC and ZL. Acquisition, analysis, and interpretation of data: XW, WW, and XG. Drafting and revising of the article: MC and ZL. Final approval of the manuscript and agreement to be accountable for all aspects of the work: all authors. All authors contributed to the article and approved the submitted version.

## Funding

This work is supported by Grants from Natural Science Foundation of Chongqing (cstc2020jcyj-msxmX0227).

## Conflict of Interest

The authors declare that the research was conducted in the absence of any commercial or financial relationships that could be construed as a potential conflict of interest.

## Publisher’s Note

All claims expressed in this article are solely those of the authors and do not necessarily represent those of their affiliated organizations, or those of the publisher, the editors and the reviewers. Any product that may be evaluated in this article, or claim that may be made by its manufacturer, is not guaranteed or endorsed by the publisher.

## Supplementary Material

Supplementary Figure 1
**(A, B)**.Network topology analysis for soft-thresholding powers. **(A)** the scale-free fit index, signed R^2^(Y) and the soft threshold power(X). **(B)** the mean connectivity(Y) and the soft threshold power (X). Choose β=3 for the subsequent analysis. **(C)**. The cluster dendrogram. In the figure, each limb represents one gene, and every color below represents one coexpression module. **(C, D)**.LASSO coefficient profiles of 30 prognostic genes for LUAD.

Supplementary Figure 2
**(A)**. Heat maps of the hub genes’ expression pattern, where the red to green means changes from high to low expression in TCGA and GEO. **(B)**. Distribution of multi-genes signature risk score in TCGA and GEO datasets. **(C)**. The survival status and interval of LUAD patients.

Supplementary Figure 3 Immune correlation analysis of SCIRGs in the model based on immune infiltration.

Supplementary Figure 4 Association between drug sensitivity and SCIRGs in the model.

Supplementary Figure 5
**(A, B)**.GSEA of the high and the low-risk group(GO). **(C, D)**. GSEA of the high- and low-risk groups(KEGG). **(E, F)**. The oncoPrint of high- and low-risk groups, the top 20 mutated genes and their mutational types and percentages are visualized in detail.

Supplementary Figure 6 Association of risk score with classical gene expression and stem cell index. **(A)** *EGFR*, **(B)** *ALK*, **(C)** *ROS1*, **(D)** *KRAS*, **(E)** *TP53*, **(F)** *CD274*, **(G)** DNAss, **(H)** RNAss.

Supplementary Figure 7 The expression level of SCIRGs in the model in different types of tumor and normal tissues *via* Oncomine.

Supplementary Figure 8 The expression level of SCIRGs in the model from UALCAN.

Supplementary Figure 9 Kaplan–Meier curves compare the OS time of the SCIRGs subgroups in LUAD.

Supplementary Figure 10 The correlation between these key genes and *EGFR*.

Supplementary Figure 11 The correlation between these key genes and *TP53*.

Supplementary Figure 12 The correlation between these key genes and CD274.

Supplementary Figure 13 the expression levels of SCIRGs in the model between Beas-2B, A549 cell lines, A549 cancer stem cell, results of the RT-PCR to determine gene expression.

Supplementary Table 1 The RT-PCR primers sequences of SCIRGs in the model.

## Abbreviations

NSCLC, non–small cell lung cancer; LUAD, lung adenocarcinoma; CSC, cancer stem cells; TCGA, The Cancer Genome Atlas; GEO, Gene Expression Omnibus; mRNAsi, mRNA stemness index; WGCNA, Weighted gene coexpression network analysis; GO, Gene Ontology; KEGG, Kyoto Encyclopedia of Genes and Genomes; GSEA, gene set enrichment analysis; TOM, topological overlap matrix; RT-PCR, reverse transcription polymerase chain reaction; DMEM, Dulbecco’s modified Eagle medium; CC, cellular component (CC); MF, molecular function; BP, biological process; DEGs, differential expression genes; TMB, tumor mutation burden; TIME, tumor immune microenvironment; SCIRGs, stem cell and immune-related differential genes; C-index, concordance index.
