# Supplementary figures and images for: Immune- and Stemness-Related Genes Revealed by Comprehensive Analysis and Validation for Cancer Immunity and Prognosis and Its Nomogram in Lung Adenocarcinoma
Source: Front Immunol. 2022 Jun 27;13:829057. doi: 10.3389/fimmu.2022.829057 (PMC9271778; doi:10.3389/fimmu.2022.829057)

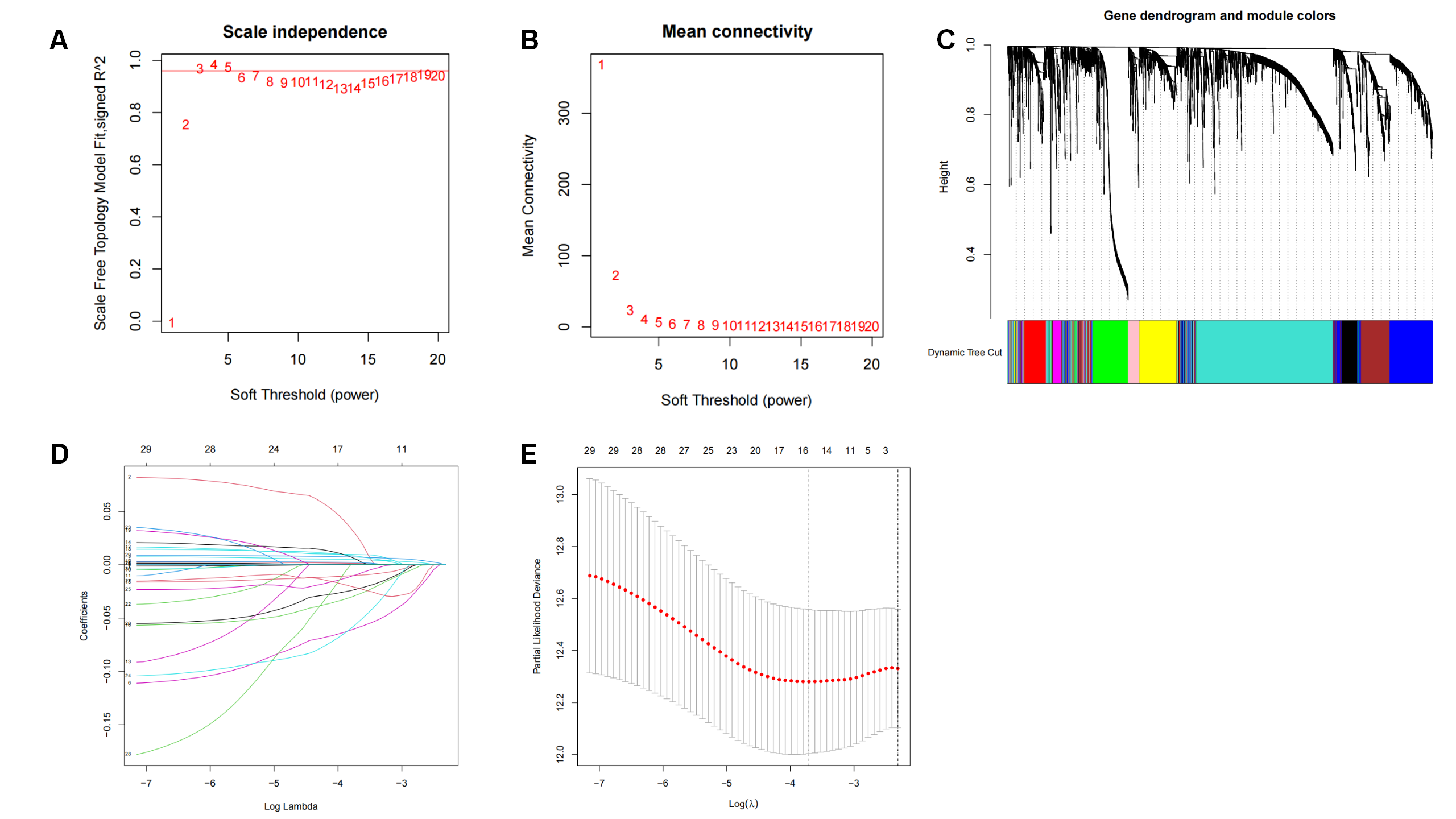

Supplement: Supplementary Figure 1 — (A, B).Network topology analysis for soft-thresholding powers. (A) the scale-free fit index, signed R2(Y) and the soft threshold power(X). (B) the mean connectivity(Y) and the soft threshold power (X). Choose β=3 for the subsequent analysis. (C). The cluster dendrogram. In the figure, each limb represents one gene, and every color below represents one coexpression module. (C, D).LASSO coefficient profiles of 30 prognostic genes for LUAD. [file Image_1.tif]

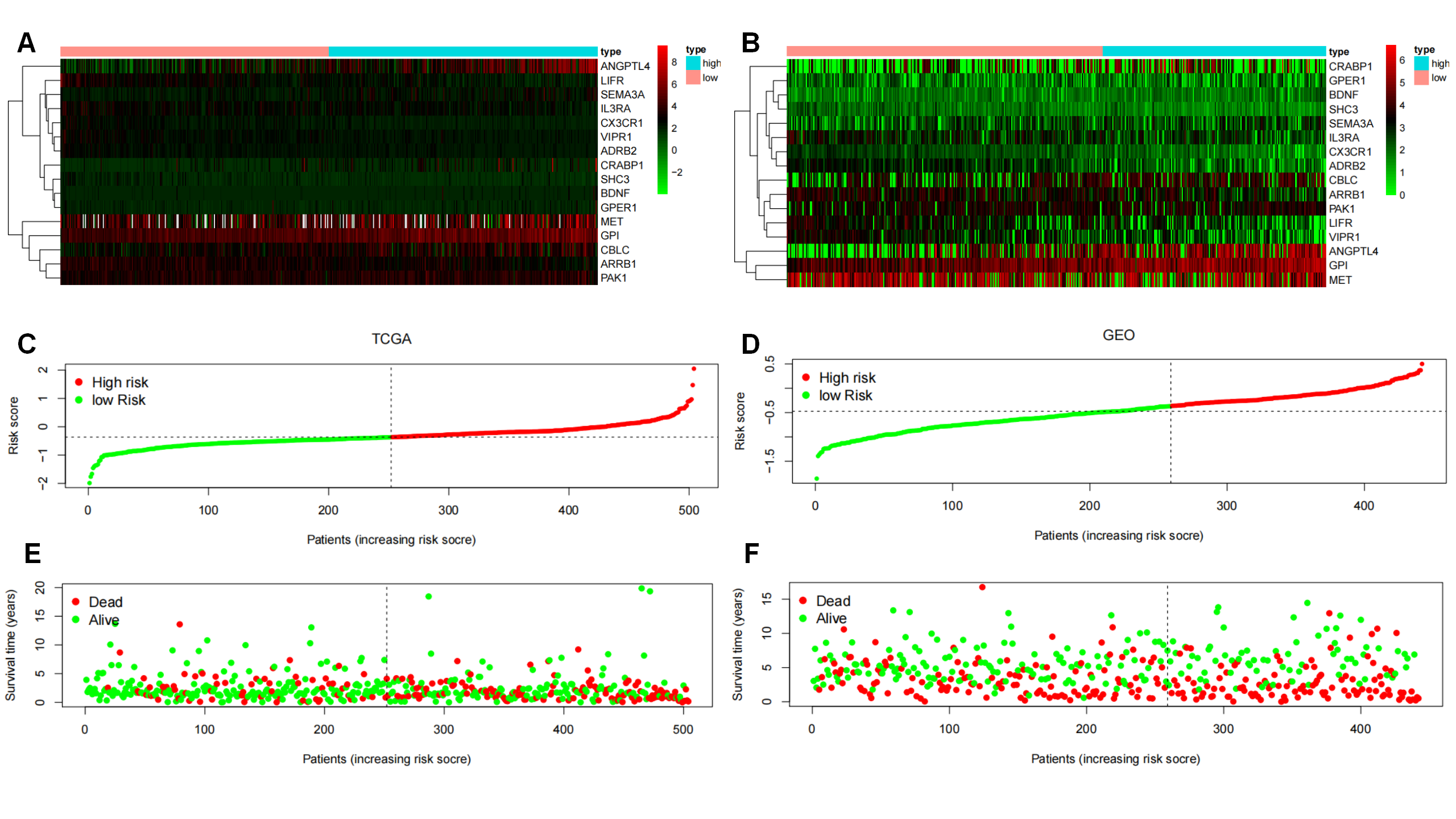

Supplement: Supplementary Figure 2 — (A). Heat maps of the hub genes’ expression pattern, where the red to green means changes from high to low expression in TCGA and GEO. (B). Distribution of multi-genes signature risk score in TCGA and GEO datasets. (C). The survival status and interval of LUAD patients. [file Image_2.tif]

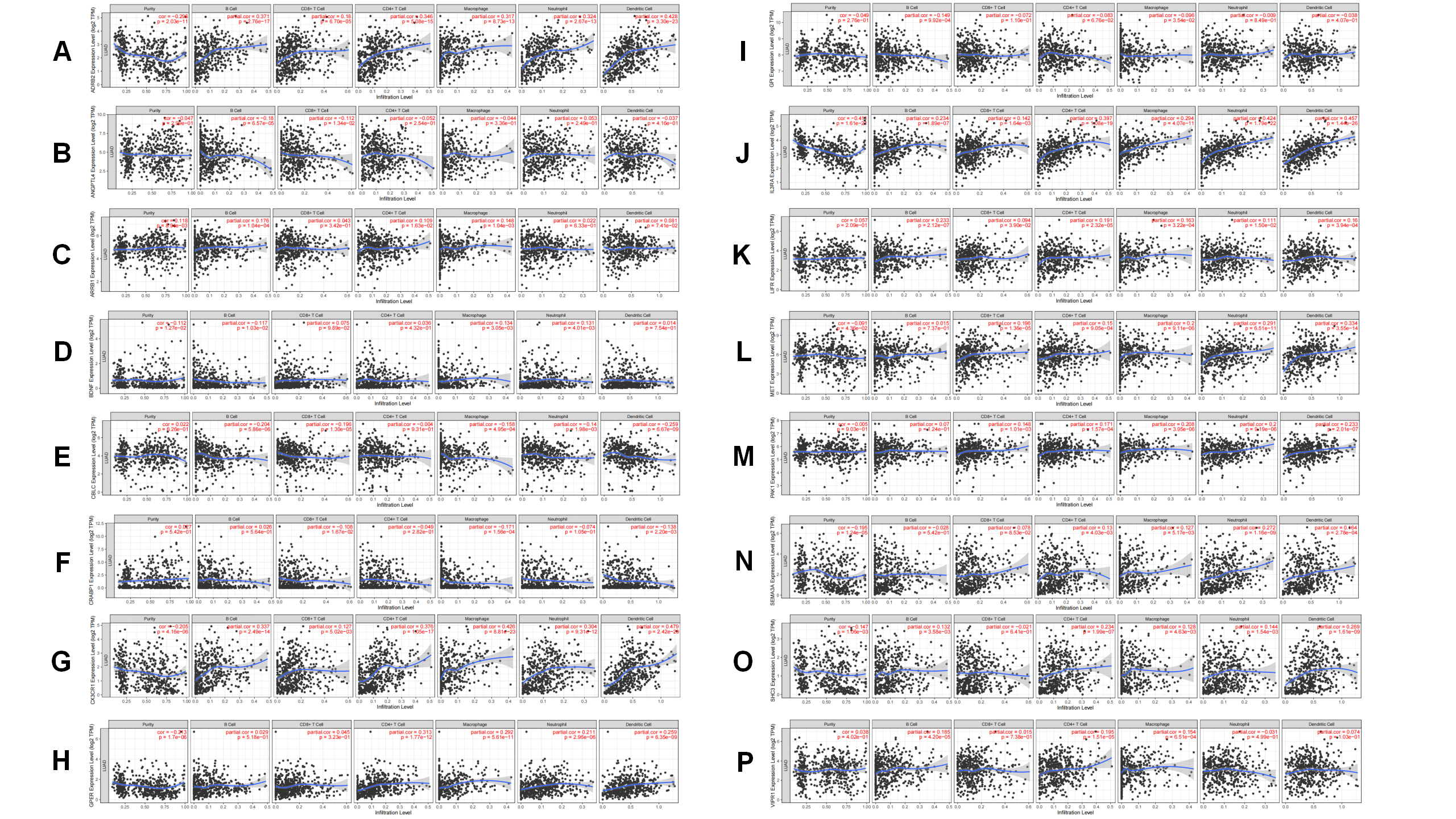

Supplement: Supplementary Figure 3 — Immune correlation analysis of SCIRGs in the model based on immune infiltration. [file Image_3.tif]

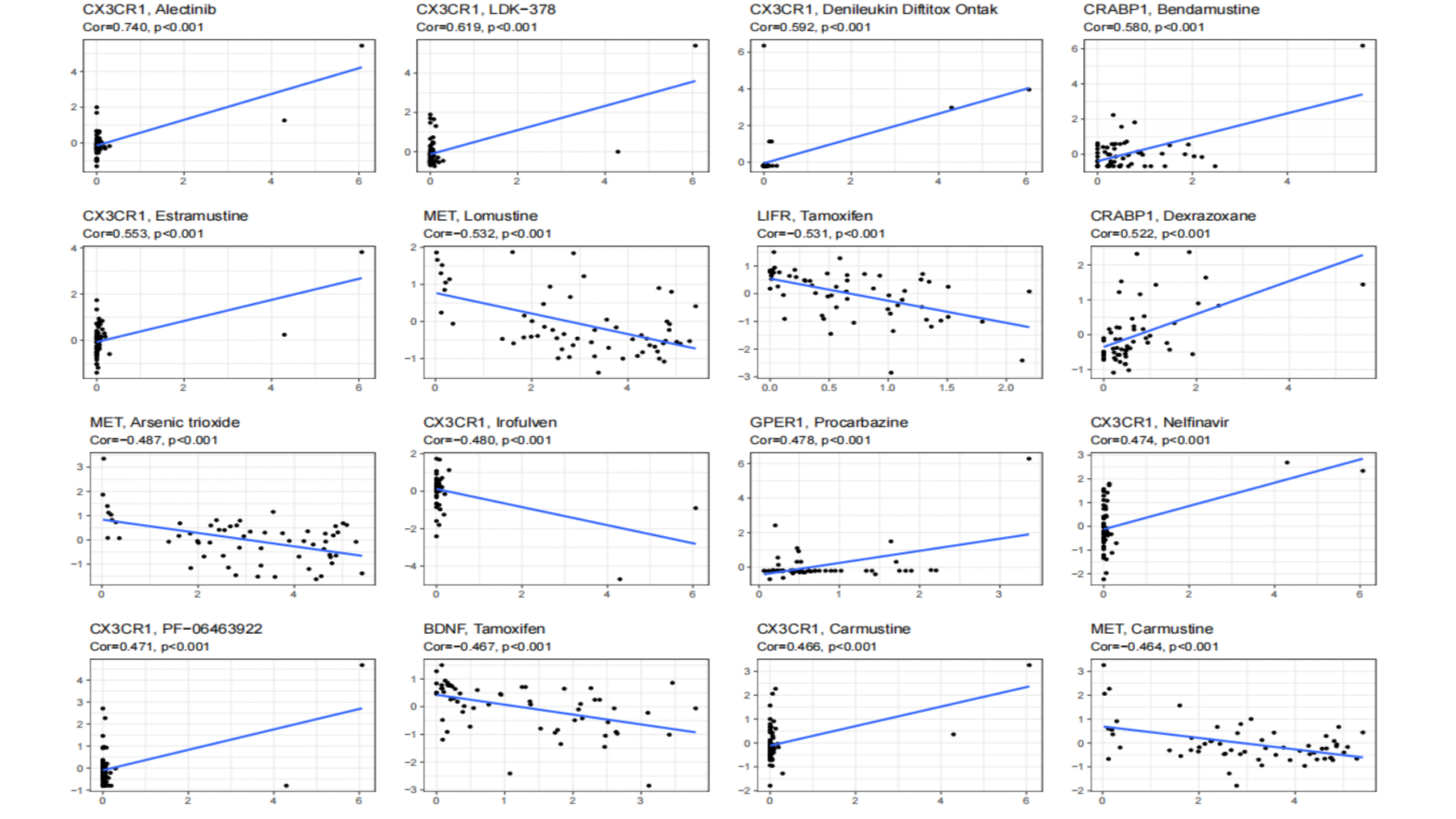

Supplement: Supplementary Figure 4 — Association between drug sensitivity and SCIRGs in the model. [file Image_4.tif]

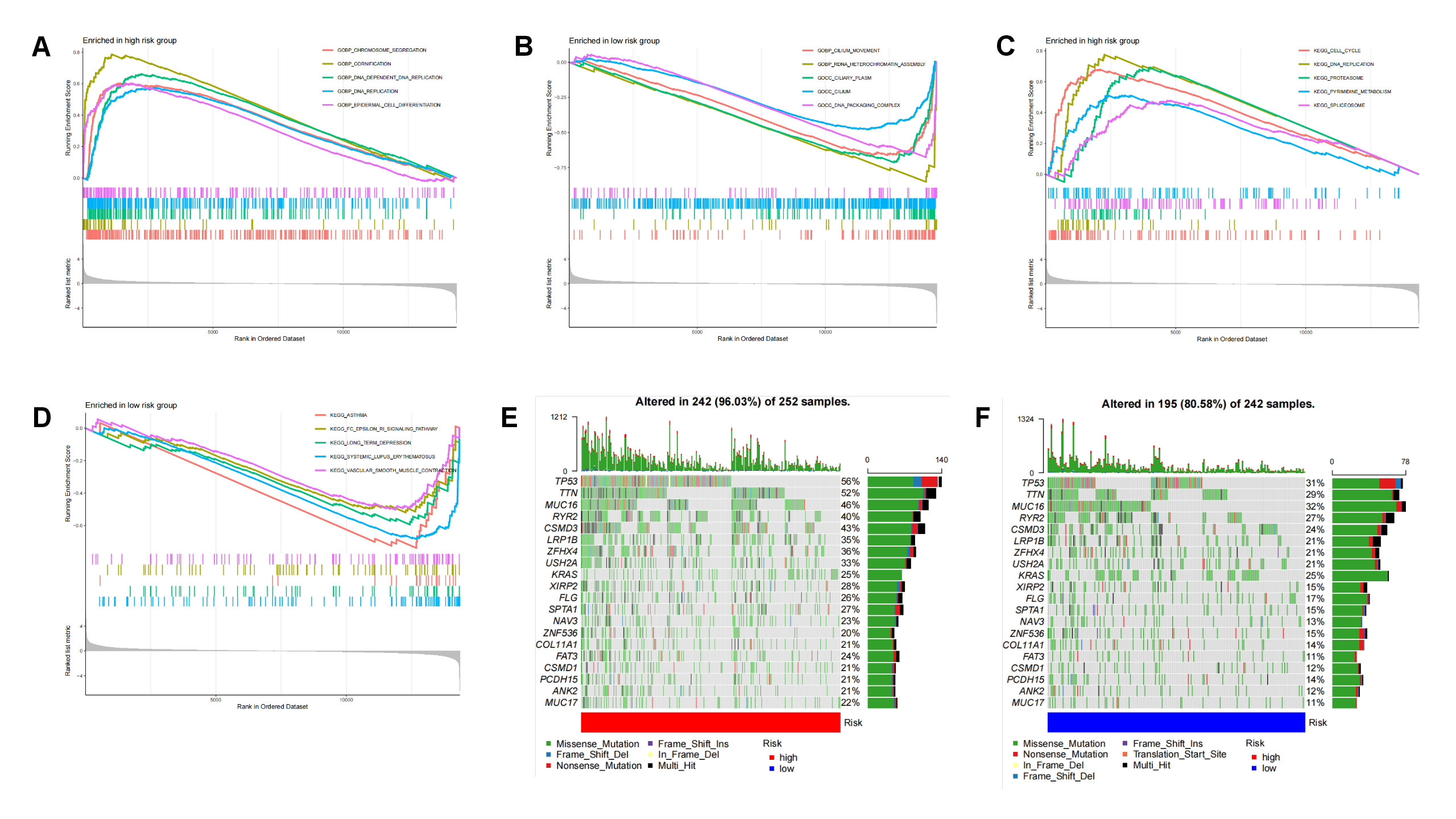

Supplement: Supplementary Figure 5 — (A, B).GSEA of the high and the low-risk group(GO). (C, D). GSEA of the high- and low-risk groups(KEGG). (E, F). The oncoPrint of high- and low-risk groups, the top 20 mutated genes and their mutational types and percentages are visualized in detail. [file Image_5.tif]

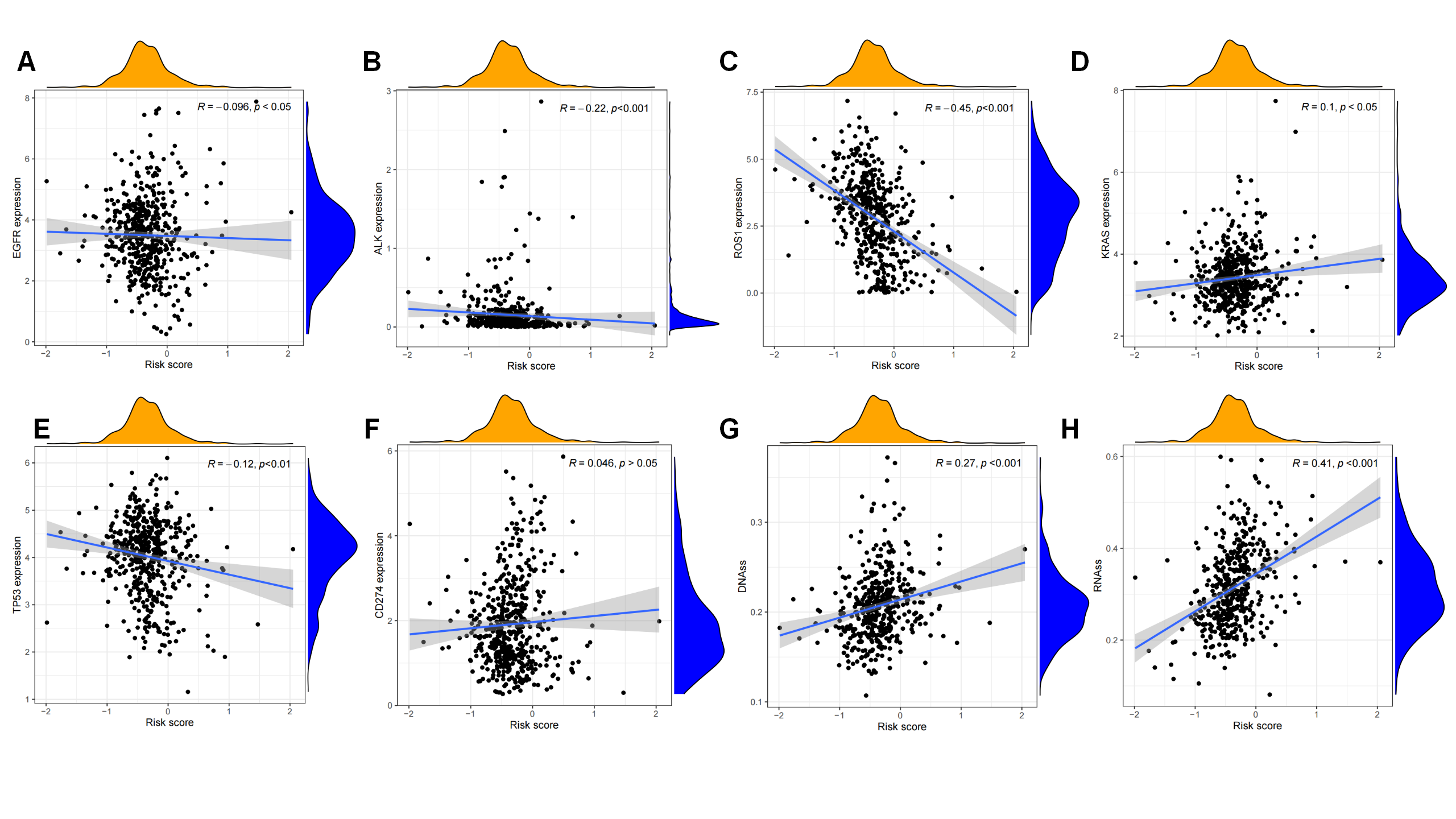

Supplement: Supplementary Figure 6 — Association of risk score with classical gene expression and stem cell index. (A) EGFR, (B) ALK, (C) ROS1, (D) KRAS, (E) TP53, (F) CD274, (G) DNAss, (H) RNAss. [file Image_6.tif]

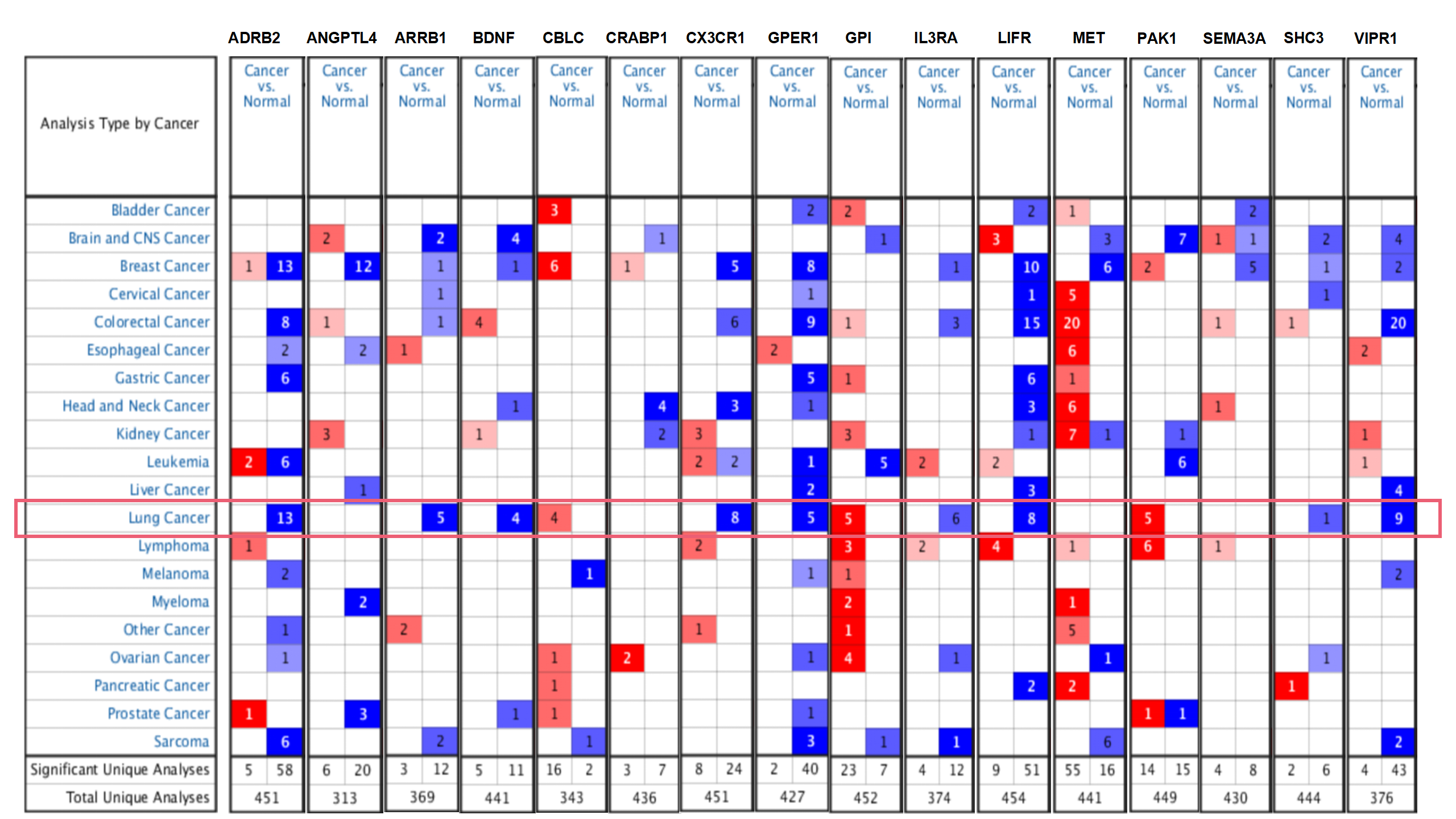

Supplement: Supplementary Figure 7 — The expression level of SCIRGs in the model in different types of tumor and normal tissues via Oncomine. [file Image_7.tif]

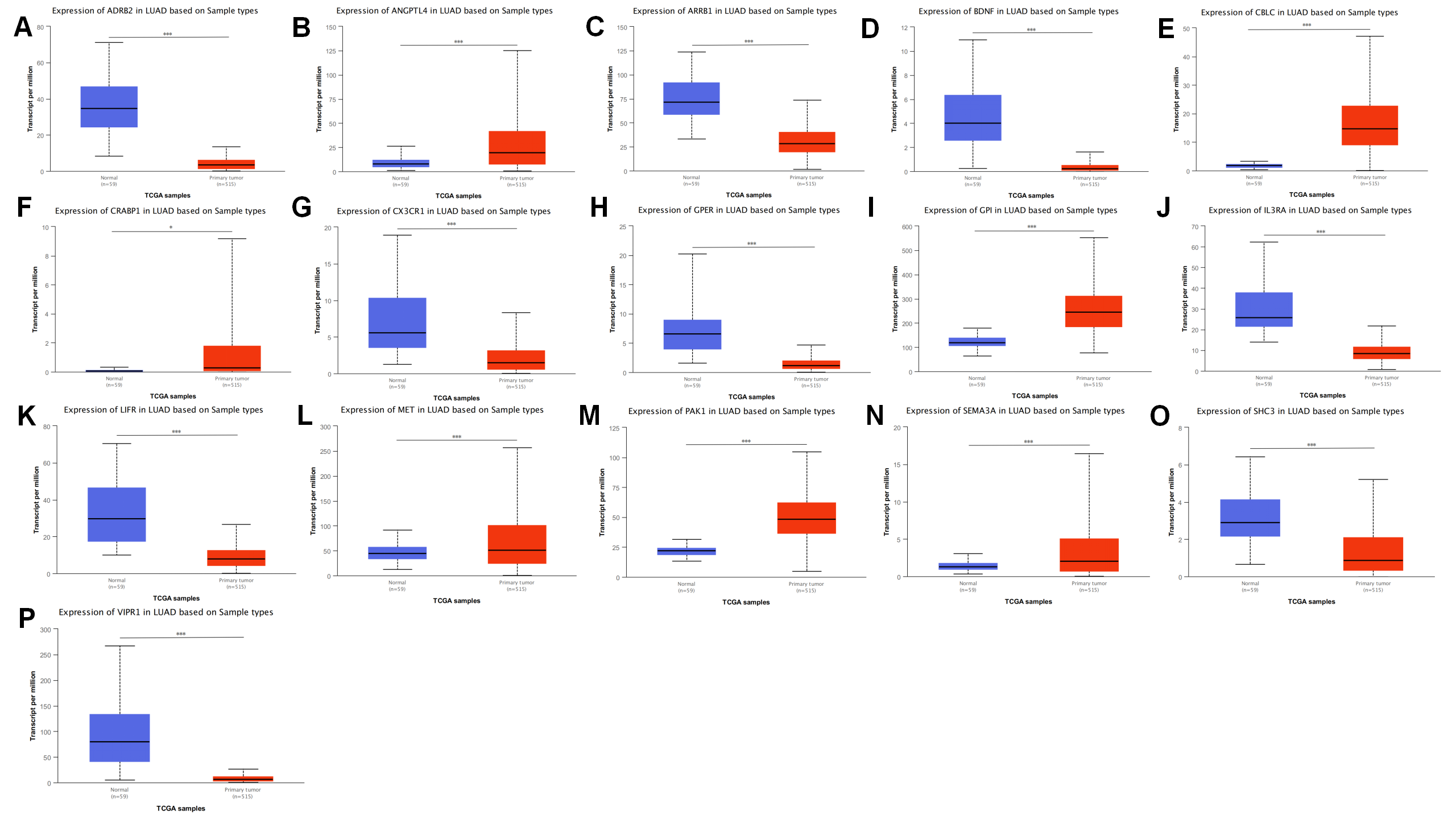

Supplement: Supplementary Figure 8 — The expression level of SCIRGs in the model from UALCAN. [file Image_8.tif]

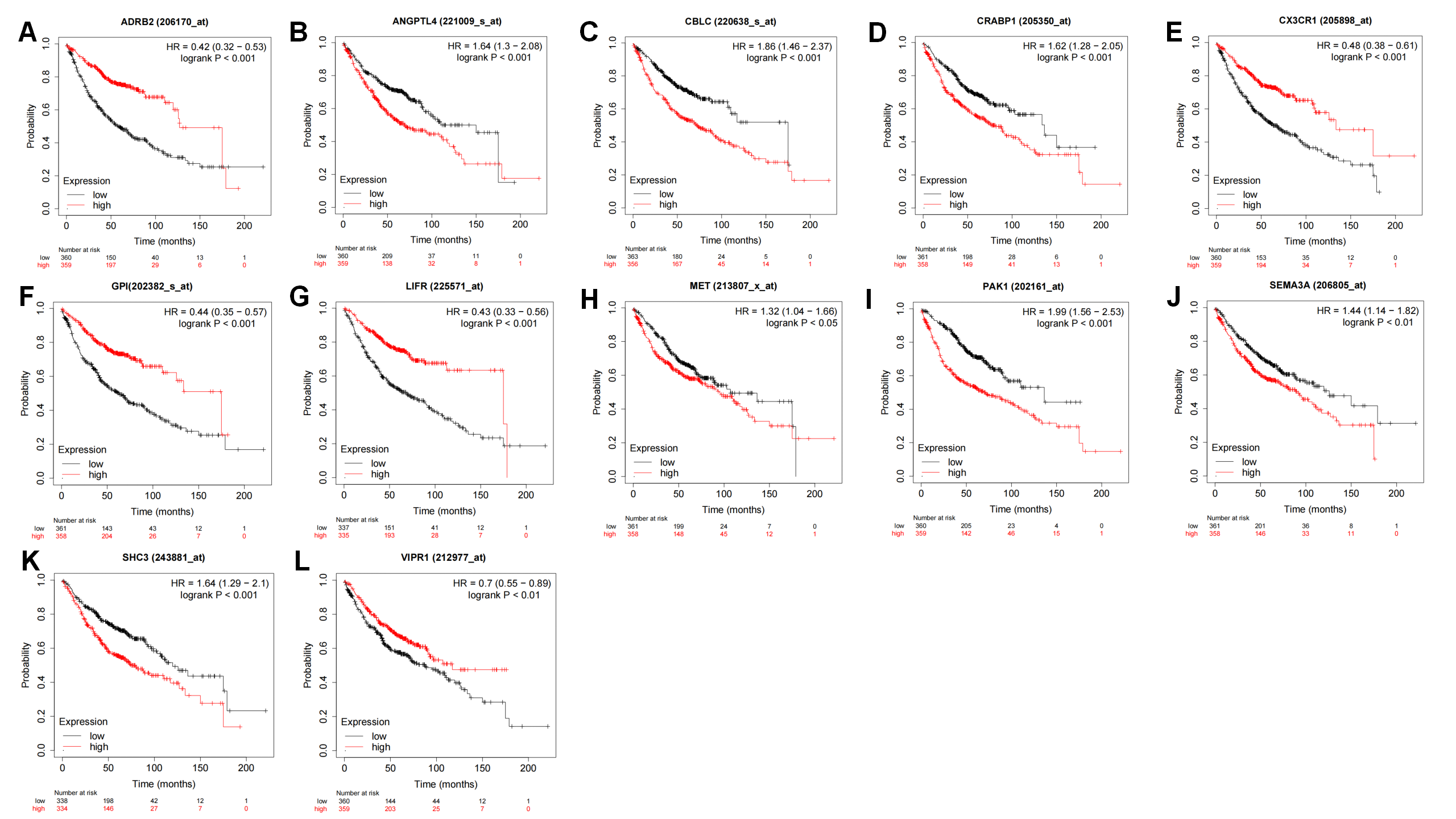

Supplement: Supplementary Figure 9 — Kaplan–Meier curves compare the OS time of the SCIRGs subgroups in LUAD. [file Image_9.tif]

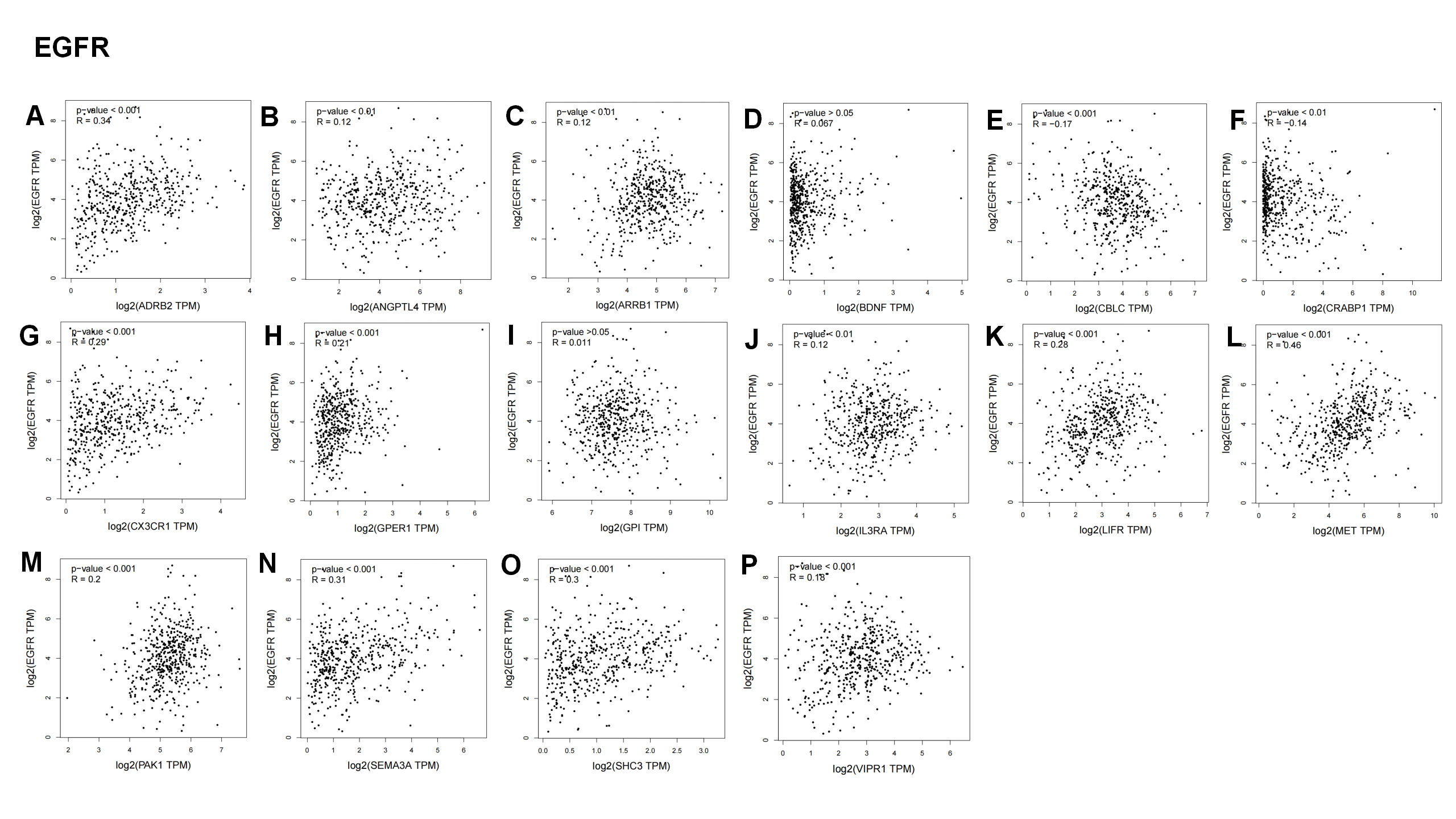

Supplement: Supplementary Figure 10 — The correlation between these key genes and EGFR. [file Image_10.tif]

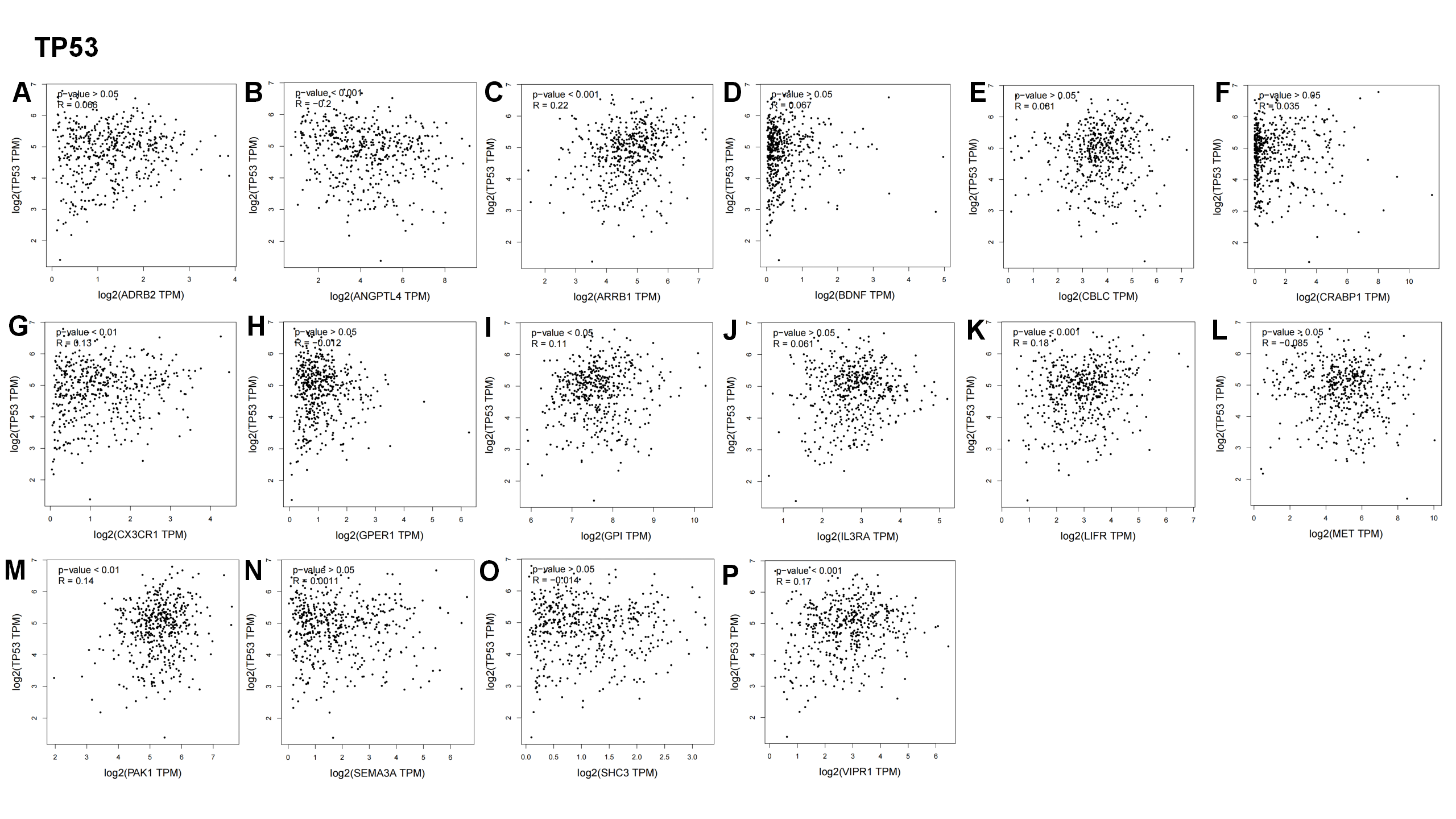

Supplement: Supplementary Figure 11 — The correlation between these key genes and TP53. [file Image_11.tif]

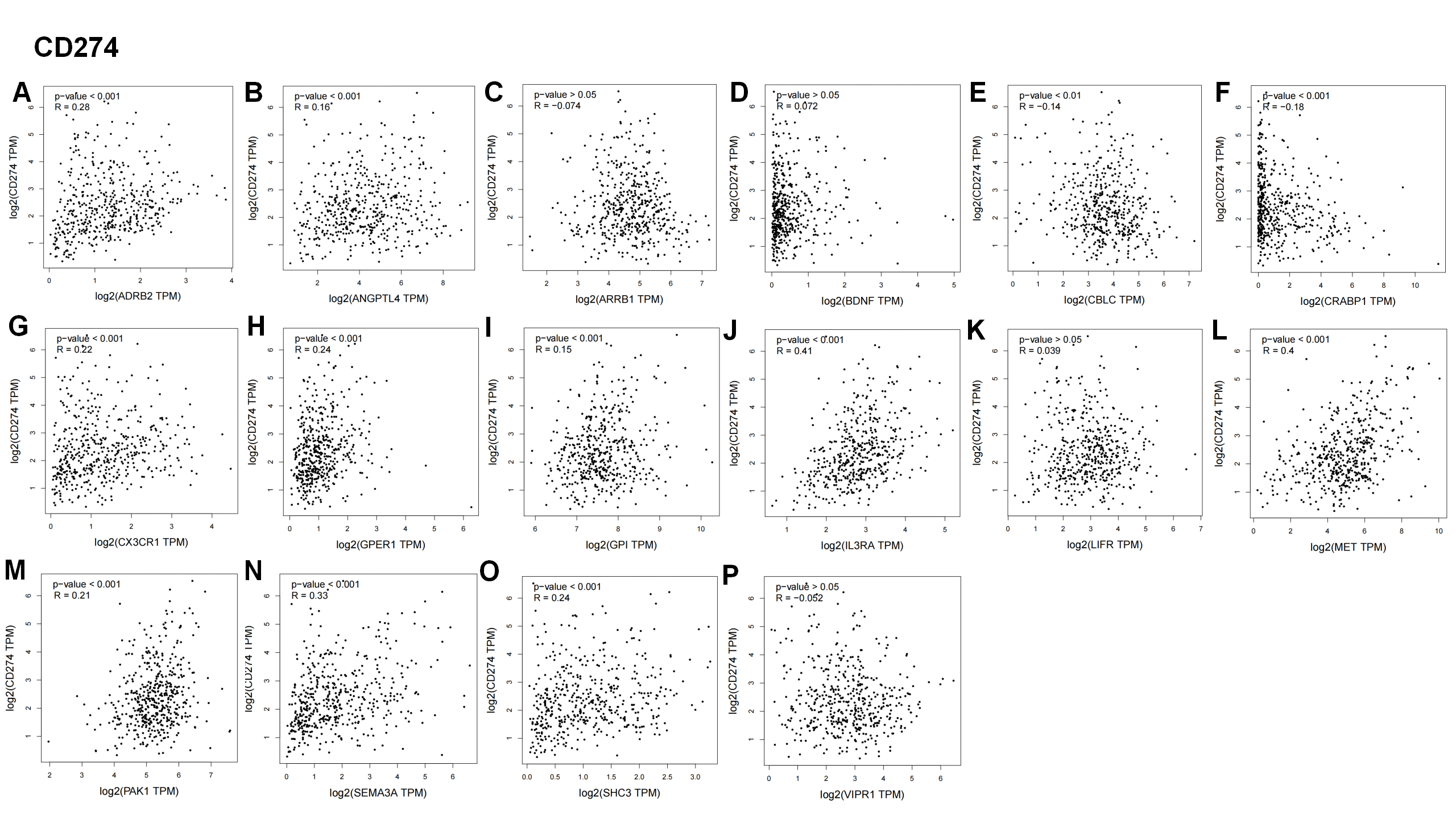

Supplement: Supplementary Figure 12 — The correlation between these key genes and CD274. [file Image_12.tif]

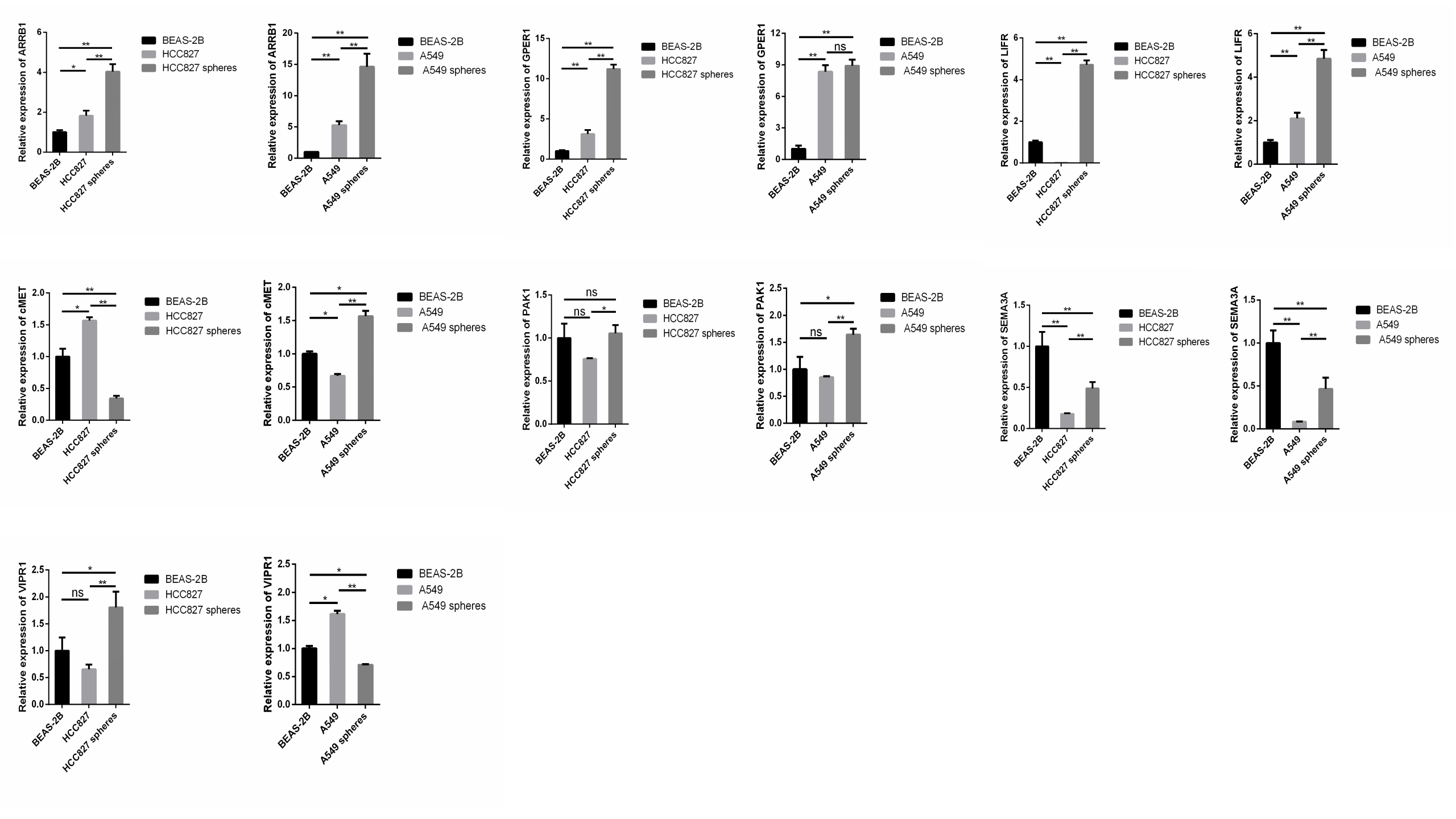

Supplement: Supplementary Figure 13 — the expression levels of SCIRGs in the model between Beas-2B, A549 cell lines, A549 cancer stem cell, results of the RT-PCR to determine gene expression. [file Image_13.tif]
