# Supplementary material for: Immune- and Stemness-Related Genes Revealed by Comprehensive Analysis and Validation for Cancer Immunity and Prognosis and Its Nomogram in Lung Adenocarcinoma
Source: Front Immunol. 2022 Jun 27;13:829057. doi: 10.3389/fimmu.2022.829057 (PMC9271778; doi:10.3389/fimmu.2022.829057)
Supplement: Supplementary Table 1 — The RT-PCR primers sequences of SCIRGs in the model. [file Table_1.docx]

| Primer Name | Base Sequence(5'to3') |
| --- | --- |
| cMET-F(human) | TAGCCAACCGAGAGACAAGC |
| cMET-R(human) | CCCACCACTGGCAAAGCAAAA |
| CX3CR1-F(human) | TAGCCACTTTGCCCTTCTGG |
| CX3CR1-R(human) | CCGATGAAGAAGAAGGCGGT |
| LIFR-F(Human) | AGTTACCACCTGGTCTTGCG |
| LIFR-R(Human) | CAGCCACTGCCAAATTTTCCT |
| PAK1-F(Human) | GATGGCACCAGAGGTTGTGA |
| PAK1-R(Human) | GCTGACAGCTTCTCTGGGTT |
| GAPDH-F(Human) | GTGGTCTCCTCTGACTTCAACA |
| GAPDH-R(Human) | CTCTTCCTCTTGTGCTCTTGCT |
| ADRB2-F(Human) | TGGGCATCGTCATGTCTCTC |
| ADRB2-R(Human) | GACGCTCGAACTTGGCAATG |
| ANGPTL4-F(Human) | CCTCTCCGTACCCTTCTCCA |
| ANGPTL4-R(Human) | AAACCACCAGCCTCCAGAGA |
| ARRB1-F(Human) | CAAAGGGACCCGAGTGTTCA |
| ARRB1-R(Human) | TTGGCCACAAACAGGTCCTT |
| BDNF-F(Human) | CAAGGCAGGTTCAAGAGGCT |
| BDNF-R(Human) | ATTGGGCCGAACTTTCTGGT |
| CBLC-F(Human) | ATGTACCAGCTCACCAAGGC |
| CBLC-R(Human) | GAGGGACTCAAACTCAGCCC |
| CRABP1-F(Human) | AGGACGGGGATCAGTTCTACA |
| CRABP1-R(Human) | AAGCCTTCTCCGACCTTGAA |
| GPER1-F(Human) | CTCTTCCCCATCGGCTTTGT |
| GPER1-R(Human) | CGGGGATGGTCATCTTCTCG |
| GPI-F(Human) | CGGACGTCATCAACATTGGC |
| GPI-R(Human) | CGGGGACCTCCTGAAGAGTA |
| IL3RA-F(Human) | CTGCAAACGAAGGAAGGTGG |
| IL3RA-R(Human) | CCTGTTGGCAACGTTCAAGT |
| SEMA3A-F(Human) | GCAGCTGATTTTATGGGGCG |
| SEMA3A-R(Human) | TGCTGCTCTGTCCTGATTGG |
| SHC3-F(Human) | ATCCCAAGCAAGATGCCTCC |
| SHC3-R(Human) | CCGAGGACCCTTGCCTTAAA |
| VIPR1-F(Human) | AGCGAGTTTGGATGAGGTGG |
| VIPR1-R(Human) | AGAAGAAGGAGACGGCAAGC |

Supplementary Table 1.
